# High-Resolution Analysis of Parent-of-Origin Allelic Expression in the Arabidopsis Endosperm

**DOI:** 10.1371/journal.pgen.1002126

**Published:** 2011-06-16

**Authors:** Philip Wolff, Isabelle Weinhofer, Jonathan Seguin, Pawel Roszak, Christian Beisel, Mark T. A. Donoghue, Charles Spillane, Magnus Nordborg, Marc Rehmsmeier, Claudia Köhler

**Affiliations:** 1 Department of Biology and Zurich-Basel Plant Science Center, Swiss Federal Institute of Technology, Zurich, Switzerland; 2 Department of Plant Biology and Forest Genetics, Uppsala BioCenter, Swedish University of Agricultural Sciences, Uppsala, Sweden; 3 Department Biosystems Science and Engineering, Swiss Federal Institute of Technology, Basel, Switzerland; 4 Genetics and Biotechnology Lab, Botany and Plant Science, National University of Ireland Galway, Aras de Brun, Ireland; 5 Gregor Mendel Institute of Molecular Plant Biology GmbH, Vienna, Austria; National Institute of Genetics, Japan

## Abstract

Genomic imprinting is an epigenetic phenomenon leading to parent-of-origin specific differential expression of maternally and paternally inherited alleles. In plants, genomic imprinting has mainly been observed in the endosperm, an ephemeral triploid tissue derived after fertilization of the diploid central cell with a haploid sperm cell. In an effort to identify novel imprinted genes in *Arabidopsis thaliana*, we generated deep sequencing RNA profiles of F1 hybrid seeds derived after reciprocal crosses of Arabidopsis Col-0 and Bur-0 accessions. Using polymorphic sites to quantify allele-specific expression levels, we could identify more than 60 genes with potential parent-of-origin specific expression. By analyzing the distribution of DNA methylation and epigenetic marks established by Polycomb group (PcG) proteins using publicly available datasets, we suggest that for maternally expressed genes (MEGs) repression of the paternally inherited alleles largely depends on DNA methylation or PcG-mediated repression, whereas repression of the maternal alleles of paternally expressed genes (PEGs) predominantly depends on PcG proteins. While maternal alleles of MEGs are also targeted by PcG proteins, such targeting does not cause complete repression. Candidate MEGs and PEGs are enriched for cis-proximal transposons, suggesting that transposons might be a driving force for the evolution of imprinted genes in Arabidopsis. In addition, we find that MEGs and PEGs are significantly faster evolving when compared to other genes in the genome. In contrast to the predominant location of mammalian imprinted genes in clusters, cluster formation was only detected for few MEGs and PEGs, suggesting that clustering is not a major requirement for imprinted gene regulation in Arabidopsis.

## Author Summary

Genomic imprinting poses a violation to the Mendelian rules of inheritance, which state functional equality of maternally and paternally inherited alleles. Imprinted genes are expressed dependent on their parent-of-origin, implicating an epigenetic asymmetry of maternal and paternal alleles. Genomic imprinting occurs in mammals and flowering plants. In both groups of organisms, nourishing of the progeny depends on ephemeral tissues, the placenta and the endosperm, respectively. In plants, genomic imprinting predominantly occurs in the endosperm, which is derived after fertilization of the diploid central cell with a haploid sperm cell. In this study we identify more than 60 potentially imprinted genes and show that there are different epigenetic mechanisms causing maternal and paternal-specific gene expression. We show that maternally expressed genes are regulated by DNA methylation or Polycomb group (PcG)-mediated repression, while paternally expressed genes are predominantly regulated by PcG proteins. From an evolutionary perspective, we also show that imprinted genes are associated with transposons and are more rapidly evolving than other genes in the genome. Many MEGs and PEGs encode for transcriptional regulators, implicating important functional roles of imprinted genes for endosperm and seed development.

## Introduction

Genomic imprinting is an epigenetic phenomenon present in mammals and flowering plants that leads to differential expression of alleles of the same gene dependent on the parent-of-origin. Imprinted genes are differentially marked in the gametes, making maternal and paternal alleles functionally different [1]. Whereas in mammals imprinting occurs in the placenta as well as the embryo and tissues of the adult organism, most examples of imprinted genes in plants to date are confined to the endosperm [2]. Although examples of imprinted genes in the plant embryo exist [3], they seem to be rare. The endosperm is a functional analog of the mammalian placenta and serves to support embryo growth [4]. It is a triploid tissue that is derived after fertilization of the homodiploid central cell with a haploid sperm cell, whereas the second sperm cell will fertilize the haploid egg cell, leading to the formation of the diploid embryo [5].

Genome-wide studies of DNA methylation in embryo and endosperm have revealed transposon and repeat sequences to be largely hypomethylated in the endosperm compared to the embryo [6], [7], with virtually all CG sequences methylated in the embryo having reduced methylation levels in the endosperm [7]. Methylation levels at CG sites are partially restored in the endosperm of mutants where the DNA glycosylase *DEMETER* (*DME*) is disrupted [7], implicating DME to be largely responsible for genome-wide CG demethylation in the endosperm. Repression of the DNA methyltransferase *MET1* in the central cell is also considered to contribute to the establishment of differential DNA methylation in the endosperm [8]. Transposon insertions or local sequence duplications are known to recruit DNA methylation and to initiate silencing of neighbouring genes in vegetative tissues [9]. This process is likely to render the targeted genes to be imprinted, as the maternal alleles can escape silencing by DME-mediated DNA demethylation in the endosperm. Based on the idea that DNA demethylation will activate genes in the endosperm that are silenced in vegetative tissues, Gehring and colleagues identified five novel imprinted genes and predicted around 50 imprinted genes in Arabidopsis, with many such genes encoding transcription factors and proteins with chromatin-related functions [6].

Although DNA methylation is widely recognized as a major mechanism for imprinted gene regulation, there are several examples suggesting that DNA methylation is not in all cases sufficient to establish imprinted gene expression. For instance, silencing of the maternal alleles of *PHERES1* (*PHE1*) and the paternal alleles of *MEDEA* (*MEA*) and *ARABIDOPSIS FORMIN HOMOLOGUE 5* (*AtFH5*) depend on repressive activity of the FERTILIZATION INDEPENDENT SEED (FIS) Polycomb group (PcG) complex [10]–[14]. The FIS PcG complex is a chromatin modifying complex that by trimethylating its target genes on histone H3 at lysine 27 (H3K27me3) causes gene repression [15]. MEA itself is a subunit of the FIS PcG complex and autoregulates its expression by repressing the paternal *MEA* allele [11]–[13], whereas activity of the maternal *MEA* allele requires DME-mediated DNA demethylation [16], [17]. Similarly, imprinted expression of *PHE1* depends on both, the FIS PcG complex and DME-mediated DNA demethylation [6], [18], [19]. Demethylation of a helitron remnant located 2.5 kbps downstream of the *PHE1* locus as well as binding of the FIS PcG complex to the *PHE1* promoter region are required for silencing of the maternal *PHE1* alleles, suggesting long-range interactions between the repeat region and PcG proteins [19].

As demethylation of repeat elements and transposons in the endosperm is a major mechanism giving rise to imprinted gene expression, it has been proposed that imprinting arose as a by-product of a silencing mechanism targeting invading foreign DNA [6], [7], [20]. Another view on the evolution of imprinted genes states that imprinting arose as a consequence of a conflict over the distribution of resources from the mother to the offspring [21], [22]. This theory predicts that there will be a selection for paternally active genes that can maximize the transfer of nutrients to the developing embryo, whereas the mother protects herself against the demands of the embryo by suppressing the growth induced by the paternally active genes. In line with this theory, imprinting occurs in placental mammals and flowering plants, both contributing maternal resources to the progeny. Furthermore, many imprinted genes in mammals affect both the demand and supply of nutrients across the placenta [23]. From the few known imprinted genes in plants, some do affect endosperm growth [24]–[26] and there is evidence that some imprinted genes may be fast evolving [27]–[29]. This suggests that imprinted gene expression, although being a likely by-product of a genome defence mechanism, may confer a selective advantage.

The discussion about the origin and evolution of imprinted gene expression in plants has been restricted by the sparse knowledge of imprinted loci. In this study we report on the identification of more than 60 genes in Arabidopsis with predicted parent-of-origin specific expression, greatly extending the number of potential imprinted loci in plants. Our study also revealed that specifically maternally and paternally expressed genes are regulated by different molecular mechanisms that rely on DNA methylation and FIS PcG function, respectively. Finally, we find that imprinted genes in plants are more rapidly evolving when compared to all other genes in the genome, and we propose that transposons may have been a driving force for the evolution of imprinted gene expression in Arabidopsis.

## Results

### Genome-Wide Identification of Genes with Parentally-Biased Expression

**Figure 1:**
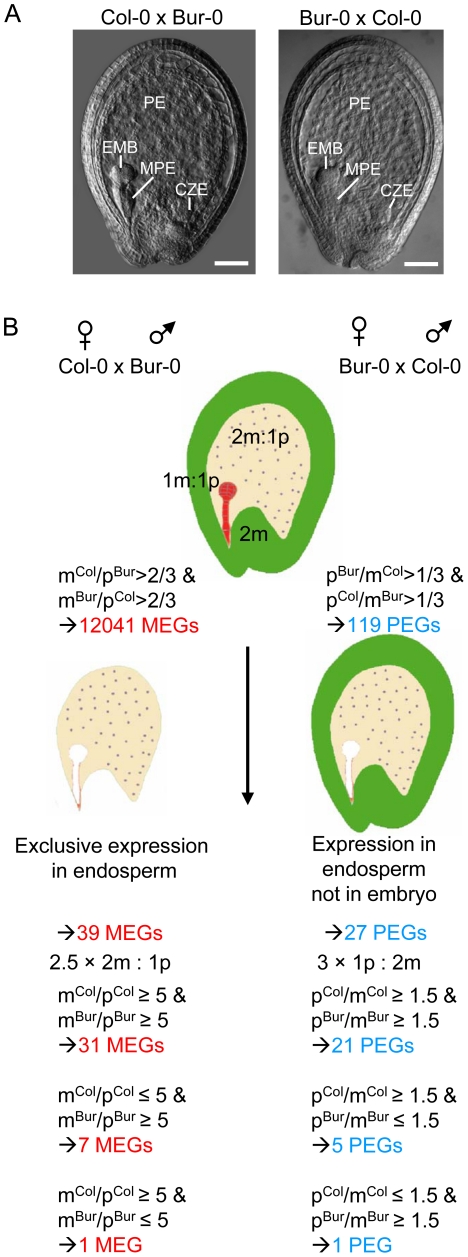
Scheme of Experimental Procedures Leading to the Identification of MEGs and PEGs in Arabidopsis. (A) Seeds derived after reciprocal crosses of Col-0 and Bur-0 accessions are phenotypically indistinguishable. Embyro (EMB), chalazal (CZE), micropylar (MPE) and peripheral (PE) endosperm are indicated. Scale bars, 50 µM. (B) Outline of filtering procedures leading to the identification of MEGs and PEGs.

We performed reciprocal crosses of the two *Arabidopsis thaliana* accessions Col-0 and Bur-0 that offer a sufficiently high number of small nucleotide polymorphisms (SNPs) to define the parent-of-origin expression of the majority of genes [30]. Seeds containing globular stage embryos were harvested at 4 days after pollination (DAP). Microscopic analysis of seeds derived from four siliques developing after reciprocal crosses of Col-0 and Bur-0 accessions did not reveal obvious developmental differences (Figure 1A), suggesting that Col-0 and Bur-0 accessions have similar properties when used as maternal plant or pollen donor. We generated mRNA-sequencing libraries of seeds derived from Col-0 × Bur-0 and Bur-0×Col-0 crosses, which we sequenced to 80-fold and 67-fold transcriptome coverage, respectively (see Materials and Methods). We identified 12041 genes (q<0.05) with maternally biased expression (maternally expressed genes, MEGs; Table S1) and 119 genes (q<0.05) with paternally biased expression (paternally expressed genes, PEGs; Table S2; see Materials and Methods for details). Within this dataset we identified seven MEGs and six PEGs that were previously predicted to be regulated by genomic imprinting [6]. We also identified the known imprinted genes *FWA* [31], *MYB3R2* [6], and *At3g25260* [6] in our MEG dataset as well as *PHE1* [10] and *At5g62110* [6] in the PEG dataset. Several known imprinted genes were not identified either due to low numbers of sequence reads (*MEA* and *FIS2*), lack of SNPs between Col-0 and Bur-0 (*MPC* [26]), or lack of significant q values (*ATFH5*, *HDG3*, *HDG8*, and *HDG9* [6]. Several of the previously identified imprinted genes (*HDG3*, *HDG8*, *HDG9*) had significantly deviating read numbers from a predicted 2m∶1p ratio only in one direction of the cross but not in the reciprocal cross and therefore failed to pass our significance threshold. This suggests that genomic imprinting can be accession-dependent and many genes may be imprinted in one accession but biallelically expressed in another accession, as was previously described for different alleles of the maize *R* and *dzr1* loci [32]–[33]. Together, based on SNP distributions deviating from the expected 2m∶1p genome ratio we successfully identified six out of twelve previously identified imprinted genes, indicating that using this approach we can successfully identify novel imprinted genes on a genome-wide scale.

### Identification of Imprinted Genes in the Endosperm

Genes that are regulated by genomic imprinting are expected to be expressed in zygotic tissues, excluding genes that contribute long lived RNAs from gametophytic tissues or that are expressed in the maternally-derived seed coat. As we used RNA isolated from seeds, many of the MEGs were likely to be seed coat-expressed genes and not necessarily imprinted genes. Therefore, we identified a stringent group of 400 genes that were preferentially expressed in the endosperm, selecting for genes with fivefold or greater signal log ratios (SLRs) in one of the endosperm domains compared with the seed coat and which were not significantly expressed in vegetative tissues (Table S3, Figure 1B and Materials and Methods). To avoid a bias towards strongly expressed genes, we included MEGs with low mRNA levels (read counts higher or equal to 10 and smaller or equal to 30) having only threefold or higher SLRs in one of the endosperm domains compared with the seed coat and which were not significantly expressed in vegetative tissues. After this selection we identified 39 candidate MEGs with maternally-biased expression (Table S4), among them *FWA*, *MYB3R2* and three predicted imprinted genes (*At2g19400*, *At3g23060*, *At4g00540* [6]). For simplicity, candidate MEGs and PEGs will be referred to as “MEGs” and “PEGs” throughout this manuscript. In contrast, MEGs and PEGs that passed additional PCR-based tests will be referred to as “confirmed MEGs and PEGs”. We considered the possibility that some MEGs are not transcribed in the endosperm but instead contribute long-lived maternally-inherited RNAs. This would predict that the mRNA levels of these genes should be reduced in seeds at 4 DAP compared to flowers containing developed female gametophytes. Out of 39 tested genes only three genes (*At1g54280*, *At3g26590*, *At4g26140*) had lower expression levels in 4 DAP seeds than in stage 12 flowers (containing eight-nucleate/seven-celled female gametophytes [34]), implicating that maternally stored mRNAs do not extensively bias the identification of imprinted genes at 4 DAP. Selection for parentally-biased expression was based on deviating sequence reads from the expected 2m∶1p ratio of maternal Col-0 (m^Col^) to paternal Bur-0 (p^Bur^) as well as maternal Bur-0 (m^Bur^) and paternal Col-0 (p^Col^) alleles. However, when analyzing the ratio of m^Col^ to p^Col^ as well as m^Bur^ to p^Col^ alleles, we noted that seven of the identified MEGs were likely to be imprinted only in the Bur-0 accession, but not in the Col-0 accession and one of the identified MEGs was likely to be imprinted only in the Col-0 accession, but not in the Bur-0 accession (Table S4), considering a fivefold higher expression of both maternal alleles over the paternal allele as maternally-biased expression. This suggests that there is considerable accession-dependency underlying the regulation of imprinted genes in Arabidopsis.

Many PEGs were strongly expressed in pollen but only weakly expressed in the endosperm (Figure S1A, S1B), implicating that transcripts loaded after fertilization from the sperm cells into the seed remained detectable in seeds at 4 DAP, as suggested by previous findings [35]. Therefore, we selected for PEGs that were present in a group of 12190 genes that we identified as being significantly expressed in the endosperm (Table S5). After this selection we obtained 38 genes (Table S6). Genes with predominant expression in the embryo are expected to mimic genes with paternally-biased expression and were excluded from the PEG list, resulting in 27 PEGs (Table S7, Figure 1B), among them previously identified genes *PHE1* [10] and *At5g62110* [6] as well as five predicted imprinted genes (*At4g11400*, *At1g48910*, *At5g50470*, *At3g19160*, *At1g23320* [6]). Considering a threefold higher expression of the paternal allele over maternal alleles as parentally-biased expression (Figure 1B), we identified five PEGs that were predominantly paternally expressed when inherited from the Col-0 parent, but biallelically expressed when inherited from Bur-0 and one PEG that was predominantly expressed when inherited from the Bur-0 parent but biallelically expressed when inherited from Col-0 (Table S7).

We considered the possibility that some of the MEGs and PEGs are regulated by parental-specific splicing. MEGs and PEGs had on average 6–8 SNPs per gene, making it rather unlikely that for genes with numbers of SNPs this large a single-exon splice variant could lead to the statistically very significant differences in overall read numbers. Nevertheless, we analyzed for every candidate gene its female and male read distributions over all SNPs of that gene over all SNPs of that gene with Pearsons's chi-square test. All MEGs and PEGs had p-values larger than 0.05, indicating that parental-specific splicing is not a major confounding factor in our analysis.

**Figure 2:**
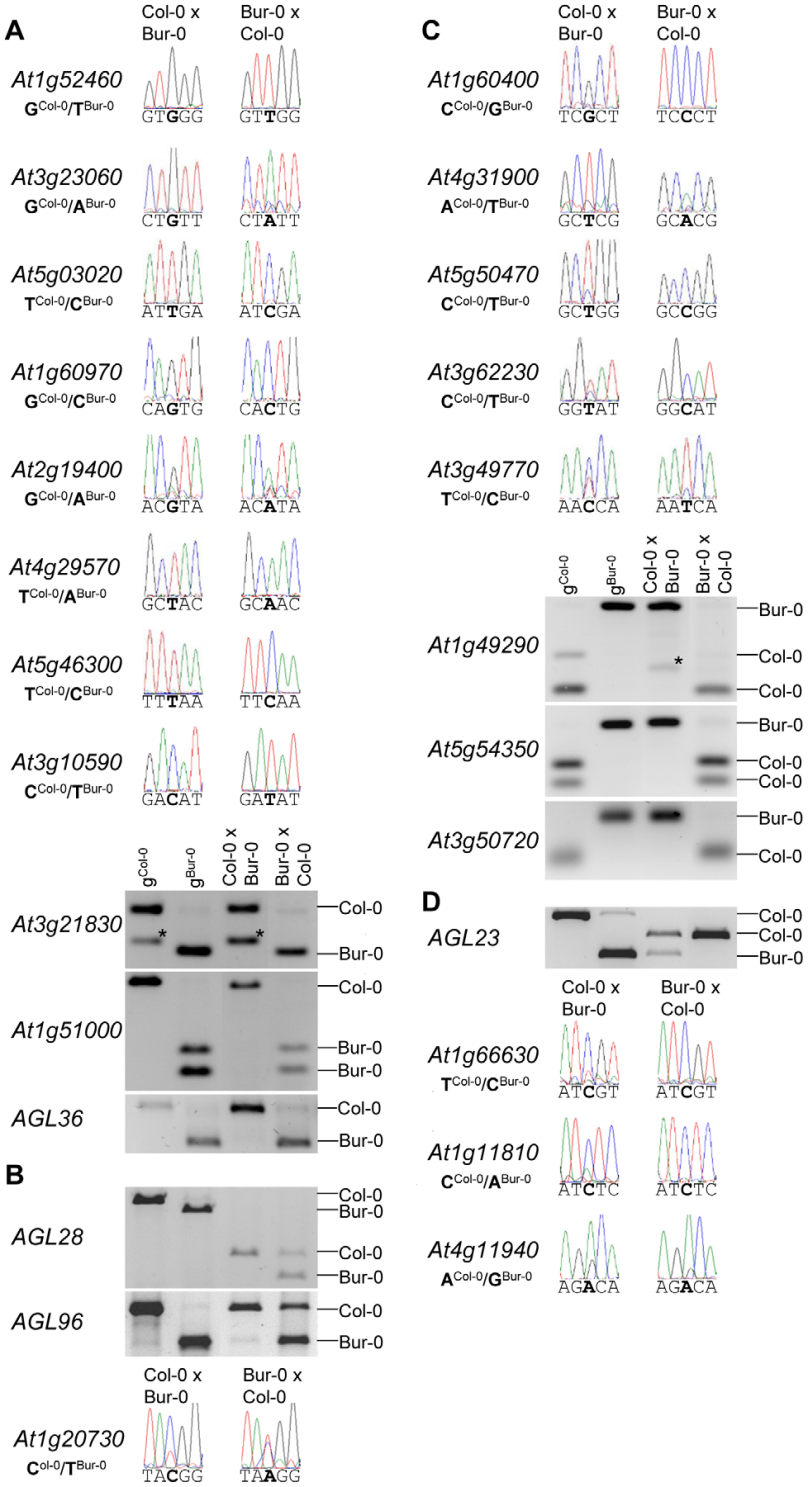
Allele-Specific Expression Analysis of MEGs and PEGs. Seeds of reciprocal crosses of Col-0 and Bur-0 accessions were harvested at 4 DAP and allele-specific expression was tested by restriction-based allele-specific PCR analysis or sequencing. MEGs and PEGs that are imprinted in both directions of the cross are shown in (A) and (C), MEGs and PEGs that are accession-dependently imprinted are shown in (B) and (D). Asterisks indicate unspecific PCR bands. Size differences between controls and cDNA samples are caused by the presence of introns in amplified regions.

We tested allele-specific expression of selected MEGs and PEGs by restriction-based allele-specific PCR analysis as well as sequencing analysis and found eleven out of twelve MEGs tested to be predominantly expressed from the maternal alleles in reciprocal crosses (*At1g52460, At3g23060, At5g03020, At1g60970, At2g19400, At4g29570, At5g46300, At3g10590*, *At3g21830*, *At1g51000*, *AGL36*; Figure 2A). One MEG, *At1g20730*, was specifically maternally expressed in the Col-0×Bur-0 cross, whereas it was biallelically expressed in the Bur-0×Col-0 cross (Figure 2B). Two MEGs with predicted accession-specific imprinting (*AGL28* and *AGL96*) were similarly regulated (Figure 2B), indicating that these three genes are exclusively maternally expressed in Bur-0, but biallelically expressed in Col-0. We also confirmed paternal-preferential expression for eight out of twelve tested PEGs (*At4g31900, At1g49290, At5g54350, At3g50720, At1g11810, At5g50470, At3g62230, At3g49770*; Figure 2C). Three of the twelve tested PEGs (*AGL23, At1g66630, At1g11810*) were preferentially paternally expressed in one direction of the cross but biallelically expressed in the reciprocal cross, indicating a significant accession-dependency in the regulation of PEGs (Figure 2D). Biallelic expression of *AGL23* in Col-0 is consistent with the previously proposed role of *AGL23* for female gametophyte development in the Col-0 accession [36]. However, our data suggest that the functional roles of *AGL23* differ between different Arabidopsis accessions. We furthermore confirmed the predicted accession-specific expression of *At4g11940*, which was paternally expressed in the cross Bur-0×Col-0, but biallelically expressed in the reciprocal cross (Figure 2D). Together, with 19 out of 24 predicted reciprocally imprinted genes being experimentally confirmed and experimental confirmation of three out of three predicted accession-dependent imprinted genes, we conclude that the majority of the newly predicted MEGs and PEGs are indeed regulated by genomic imprinting.

Among reciprocally imprinted and accession-dependent imprinted MEGs and PEGs we detected a significant enrichment of nuclear localized proteins and transcription factors (Table S8), with many of them belonging to AGL MADS-box transcription factors (AGL36, AGL28, AGL96, PHE1, AGL23) that in yeast two-hybrid studies were shown to directly or indirectly interact with each other [37] as well as with AGL62 [38] (Figure S2). AGL62 has been proposed to be a major regulator of endosperm cellularization [38], suggesting a major regulatory role of imprinting genes in timing the onset of endosperm cellularization. Furthermore, MEGs were enriched for genes encoding cytidine deaminases (p = 3 E−4) that in zebrafish as well as in mammals have been proposed to be required for DNA demethylation [39], [40]. Whether cytidine deaminases play a similar role in the plant endosperm remains to be tested.

### Dynamics of MEG and PEG Expression during Seed and Vegetative Development

**Figure 3:**
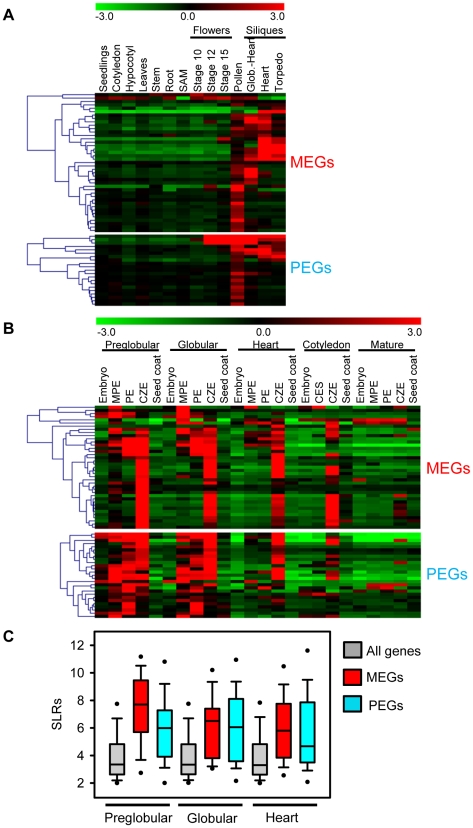
Expression Analysis of MEGs and PEGs in Vegetative and Seed Tissues. (A) Cluster analysis of MEGs and PEGs (including accession-dependent MEGs and PEGs) based on their expression in vegetative tissues and seeds. Each row represents a gene, and each column represents a tissue type. Tissue types are: seedlings, cotyledons, hypocotyl, leaves, stems, roots, shoot apical meristem (SAM), flowers at stages 10, 12, 15, siliques containing seeds with embryos in the globular to heart stage, heart stage and torpedo stage. Red or green indicate tissues in which a particular gene is highly expressed or repressed, respectively. (B) Cluster analysis of MEGs and PEGs (including accession-dependent MEGs and PEGs) based on their expression in embryo, endosperm and seed coat during different stages of seed development. Each row represents a gene, and each column represents a tissue type. Tissue types are: embryos from the preglobular stage to the mature stage, micropylar (MPE), peripheral (PE) and chalazal (CZE) endosperm derived from seeds containing embryos of the preglobular stage to the mature stage, and seed coat derived from seeds containing embryos of the preglobular stage to the mature stage. Red or green indicate tissues in which a particular gene is highly expressed or repressed, respectively. (C) Box plots of expression levels of MEGs (including accession-dependent MEGs; red) and PEGs (including accession-dependent PEGs; blue) compared to all genes (gray) in the chalazal endosperm region of seeds containing preglobular, globular and heart stage embryos. SLRs, Signal Log Ratios based on ATH1 microarray signals after RMA normalization.

Whereas MEGs were filtered based on the absence of expression in vegetative tissues, these filtering criteria were not applied for PEGs. However, expression patterns of MEGs and PEGs were very similar, both types of imprinted genes had a preference for being expressed in pollen, but were mainly excluded from vegetative tissues (Figure 3A). The expression profile of MEGs and PEGs in seeds was very comparable as well, both types of genes were not expressed in the seed coat, rarely expressed in the embryo, but most MEGs and PEGs were strongly expressed in the chalazal region of the endosperm (Figure 3B). Whereas expression of most MEGs was confined to the chalazal region of the endosperm, PEG expression was less restricted and extended to the peripheral and micropylar regions of the endosperm. MEG and PEG expression was clearly detectable in the endosperm of seeds containing preglobular stage embryos and expression in the chalazal endosperm remained detectable until seeds contained cotyledon stage embryos. Expression in the micropylar and peripheral region of the endosperm was only detectable until seeds contained heart stage embryos, after this stage expression remained confined to the chalazal endosperm. Average expression levels of MEGs and PEGs in the chalazal endosperm region were clearly above average expression levels of all genes, with expression being highest at the preglobular stage and declining towards the heart stage of seed development (Figure 3C).

### Different Localization and Regulatory Impact of DNA Methylation at MEG and PEG Loci

**Figure 4:**
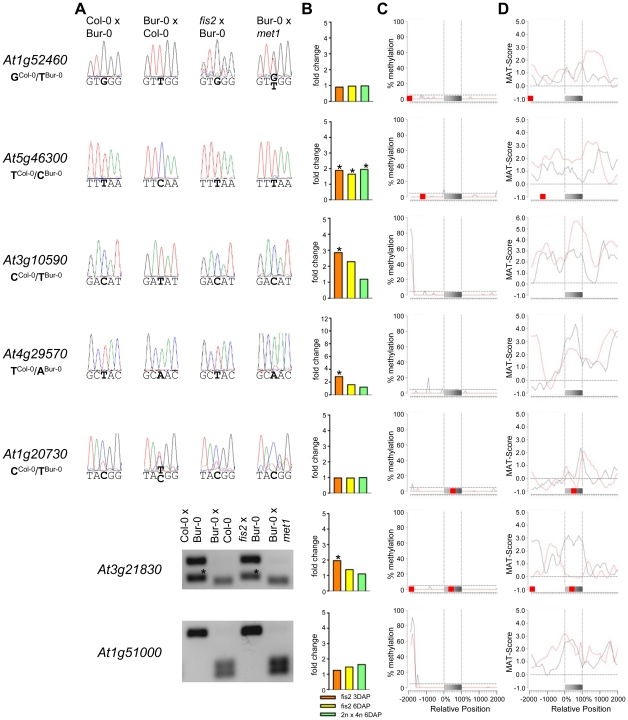
Impact of DNA Methylation and FIS PcG Function on the Regulation of Confirmed MEGs without Prominent Genic CG DNA Methylation. (A) Allele-specific expression analysis of indicated MEGs in seeds derived from crosses of Col-0×Bur-0, Bur-0×Col-0, *fis2*×Bur-0 and Col-0×*met1*. Seeds were harvested at 4 DAP and allele-specific expression was tested by restriction-based allele-specific PCR analysis or sequencing. Asterisks indicate unspecific PCR bands. (B) Fold-changes of MEG expression in *fis2* mutant seeds at 3 and 6 days after pollination (DAP) and from seeds derived from pollination with tetraploid pollen donors at 6 DAP compared to wild-type seeds at the corresponding time points. Data are based on ATH1 microarray signals after RMA normalization. Significantly deregulated genes are marked by an asterisk. (C) CG DNA methylation profiles of indicated MEGs in vegetative tissues (black line) or endosperm (red line) based on data published by [7], [41]. The gray bar represents the annotated gene body from transcription start (left) to transcription end (right). Red boxes represent transposable elements. Profiles are shown for 5% length intervals along the gene body and for 100 bp sequence intervals for the 2-kb regions upstream and downstream of each gene. The vertical dotted lines mark the gene body. The horizontal dashed line marks the DNA methylation level in vegetative tissues of TAIR8-annotated genes at the transcriptional start site. (D) H3K27me3 profiles of indicated MEGs in vegetative tissues (black line) or endosperm (red line) based on data published by [52], [64]. The gray bar represents the annotated gene body from transcription start (left) to transcription end (right). Red boxes represent transposable elements. Profiles are shown for 5% length intervals along the gene body and for 100 bp sequence intervals for the 2-kb regions upstream and downstream of each gene. The vertical dotted lines mark the gene body. The horizontal dashed line marks the H3K27me3 level of TAIR8-annotated genes at the transcriptional start site.

**Figure 5:**
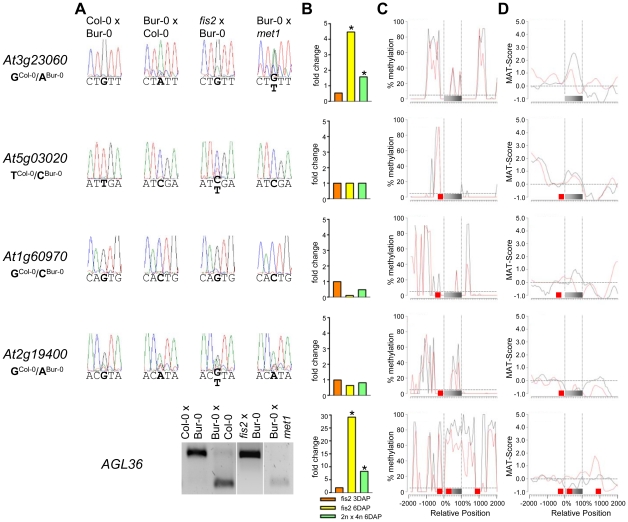
Impact of DNA Methylation and FIS PcG Function on the Regulation of Confirmed MEGs with Prominent Genic CG DNA Methylation. (A) Allele-specific expression analysis of indicated MEGs in seeds derived from crosses of Col-0×Bur-0, Bur-0×Col-0, *fis2*×Bur-0 and Col-0×*met1*. Seeds were harvested at 4 DAP and allele-specific expression was tested by restriction-based allele-specific PCR analysis or sequencing. (B) Fold-changes of MEG expression in *fis2* mutant seeds at 3 and 6 days after pollination (DAP) and from seeds derived from pollination with tetraploid pollen donors at 6 DAP compared to wild-type seeds at the corresponding time points. Data are based on ATH1 microarray signals after RMA normalization. Significantly deregulated genes are marked by an asterisk. (C) CG DNA methylation profiles of indicated MEGs in vegetative tissues (black line) or endosperm (red line) based on data published by [7], [41]. The gray bar represents the annotated gene body from transcription start (left) to transcription end (right). Red boxes represent transposable elements. Profiles are shown for 5% length intervals along the gene body and for 100 bp sequence intervals for the 2-kb regions upstream and downstream of each gene. The vertical dotted lines mark the gene body. The horizontal dashed line marks the DNA methylation level in vegetative tissues of TAIR8-annotated genes at the transcriptional start site. (D) H3K27me3 profiles of indicated MEGs in vegetative tissues (black line) or endosperm (red line) based on data published by [52], [64]. The gray bar represents the annotated gene body from transcription start (left) to transcription end (right). Red boxes represent transposable elements. Profiles are shown for 5% length intervals along the gene body and for 100 bp sequence intervals for the 2-kb regions upstream and downstream of each gene. The vertical dotted lines mark the gene body. The horizontal dashed line marks the H3K27me3 level of TAIR8-annotated genes at the transcriptional start site.

Previous studies predicted imprinted genes based on the assumption that DNA demethylation in the central cell causes activation of maternal alleles of genes, whereas paternal alleles remain methylated and silenced [6]. Whereas this assumption should predict MEGs, it is unlikely to successfully predict PEGs. However, in our PEG dataset we found five previously predicted imprinted genes [6], indicating that DNA methylation is important, but not the only regulator of imprinted gene expression. We analyzed the CG DNA methylation status of MEGs and PEGs in vegetative tissues and in the endosperm at 7–9 DAP using previously published data [7], [41]. Allele-specific DNA methylation patterns are established during gametogenesis and immediately after fertilization [42], [43], implicating that the endosperm DNA methylation profile at 7–9 DAP is similar to the profile at 4 DAP (time point of this study). There are two main classes of MEGs distinguishable based on the CG DNA methylation profile: MEGs without substantial CG DNA methylation in immediate vicinity to the genic regions (Figure 4 and Figure S3A–S3C) and MEGs with high levels of CG DNA methylation surrounding genic regions (Figure 5 and Figure S3D–S3F).

**Figure 6:**
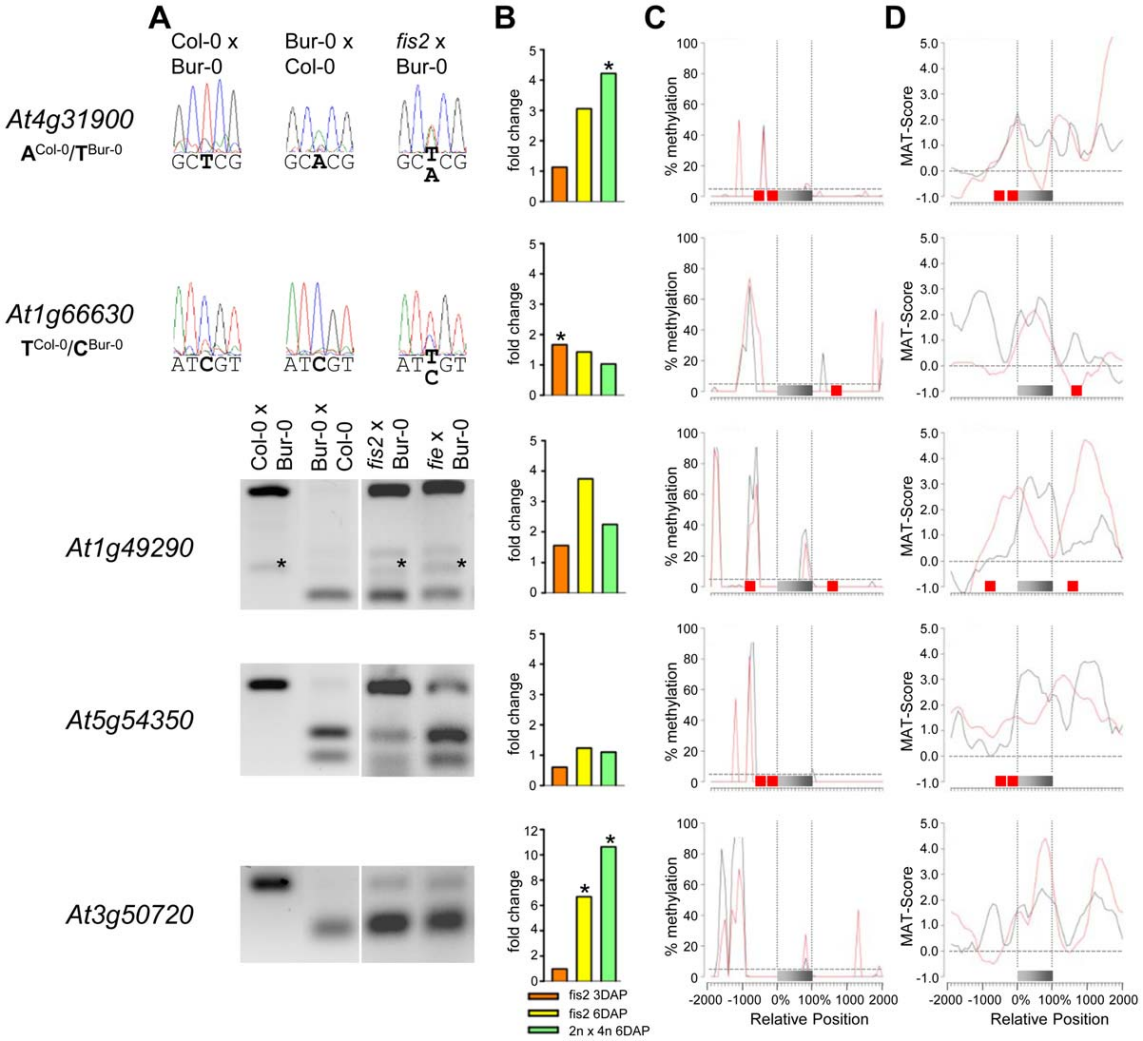
Impact of FIS PcG Function on the Regulation of Confirmed PEGs. (A) Allele-specific expression analysis of indicated PEGs in seeds derived from crosses of Col-0×Bur-0, Bur-0×Col-0, *fis2*×Bur-0, and *fie*×Bur-0. Seeds were harvested at 4 DAP and allele-specific expression was tested by restriction-based allele-specific PCR analysis or sequencing. Asterisks indicate unspecific PCR bands. (B) Fold-changes of PEG expression in *fis2* mutant seeds at 3 and 6 days after pollination (DAP) and from seeds derived from pollination with tetraploid pollen donors at 6 DAP compared to wild-type seeds at the corresponding time points. Data are based on ATH1 microarray signals after RMA normalization. Significantly deregulated genes are marked by an asterisk. (C) CG DNA methylation profiles of indicated PEGs in vegetative tissues (black line) or endosperm (red line) based on data published by [7], [41]. The gray bar represents the annotated gene body from transcription start (left) to transcription end (right). Red boxes represent transposable elements. Profiles are shown for 5% length intervals along the gene body and for 100 bp sequence intervals for the 2-kb regions upstream and downstream of each gene. The vertical dotted lines mark the gene body. The horizontal dashed line marks the DNA methylation level in vegetative tissues of TAIR8-annotated genes at the transcriptional start site. (D) H3K27me3 profiles of indicated PEGs in vegetative tissues (black line) or endosperm (red line) based on data published by [52], [64]. The gray bar represents the annotated gene body from transcription start (left) to transcription end (right). Red boxes represent transposable elements. Profiles are shown for 5% length intervals along the gene body and for 100 bp sequence intervals for the 2-kb regions upstream and downstream of each gene. The vertical dotted lines mark the gene body. The horizontal dashed line marks the H3K27me3 level of TAIR8-annotated genes at the transcriptional start site.

The CG DNA methylation profile of PEGs was clearly distinguishable from the MEG CG profiles; almost all PEGs had CG methylation peaks on average about 700 bps up- or downstream of the coding regions, whereas coding regions and the immediate up- and downstream regions were mostly devoid of CG DNA methylation (Figure 6 and Figure S4), suggesting that low levels of DNA methylation in this region are important for keeping PEGs transcriptionally active when paternally inherited and that DNA methylation at this region is unlikely to be responsible for keeping maternal alleles of PEGs silenced. The level of CG methylation in MEGs and PEGs was reduced in the endosperm providing an explanation why MEGs and PEGs were predicted in a previous study [6].

Demethylation of the paternal genome by mutations in the DNA methyltransferase *MET1* has been demonstrated to cause biallelic expression of several MEGs [26], [31], [44], [45]. Therefore, we tested allele-specific expression of confirmed MEGs in seeds derived after pollination with pollen of *met1/MET1* plants. Out of six tested MEGs that were not substantially CG methylated in the genic region, there were three genes that became either biallelically expressed upon pollination with *met1* pollen (*At1g52460*) or exclusively paternally expressed (*At5g46300*, *At3g10590*; Figure 4). The accession-dependent imprinted gene *At1g20730* which was preferentially maternally expressed in the cross Col-0×Bur-0 but biallelically expressed in the reciprocal cross became as well paternally expressed upon *met1* pollination (Figure 4). Analysis of non-CG DNA methylation revealed that within a distance of 2 kbps upstream of the transcriptional start site MEGs *At5g46300, At3g10590* and *At1g20730* were substantially marked by CHG or CHH methylation (Figure S5A), suggesting that non-CG DNA methylation might be involved in the repression of paternal MEG alleles. Non-CG DNA methylation levels were higher in the endosperm compared to vegetative tissues, in agreement with previous reports on frequent small interfering RNA–targeted hypermethylation of CHG and CHH target sites in the endosperm [7]. Out of five tested MEGs with substantial CG DNA methylation in the vicinity of genic regions only one MEG became reactivated upon pollination with *met1* pollen (*At3g23060*; Figure 5), suggesting that prominent CG DNA methylation marks are not a decisive criterion for DNA methylation-dependent repression of the paternal MEG alleles. Conversely, the absence of prominent CG DNA methylation in vicinity of genic regions does not exclude a regulatory role of DNA methylation on the activity status of paternal MEG alleles.

### MEGs and PEGs Are Regulated by the FIS PcG Complex

As DNA methylation seemed unlikely to be responsible for repression of the paternal alleles of many MEGs as well as the maternal alleles of PEGs, we further investigated by which alternative mechanism imprinting of MEGs and PEGs is regulated. Almost all PEGs (25 out of 27 PEGs and accession-dependent PEGs) and many MEGs (31 out of 39 MEGs and accession-dependent MEGs) were PcG target genes in vegetative tissues (Tables S4, S7). In the endosperm, the average H3K27me3 levels of MEGs and PEGs were clearly increased over the H3K27me3 levels of all genes, with the H3K27me3 levels of PEGs being twice as high as the H3K27me3 levels of MEGs (Figure S6), suggesting that silencing of the maternally inherited alleles of PEGs is mediated by the FIS PcG complex. This hypothesis would predict increased expression of PEGs upon loss of FIS function. We tested this hypothesis by analyzing expression levels of PEGs in *fis2* mutants at 3 DAP and 6 DAP. Indeed, half of all PEGs and accession-dependent PEGs were significantly upregulated in the *fis2* mutant (Figure 6 and Figure S4). Furthermore, we tested allele-specific expression of four confirmed PEGs (*At4g31900*, *At1g49290*, *At5g54350*, *At3g50720*) and one accession-dependent confirmed PEG (*At1g66630*) in *fis2* and *fie* mutants lacking maternal FIS function. For all four PEGs as well as the accession-dependent PEG loss of FIS function caused activation of maternal PEG alleles, adding further support to our hypothesis (Figure 6).

Also 13 out of 39 MEGs and accession-dependent MEGs were significantly upregulated in the *fis2* mutant (Figure 4, Figure 5 and Figure S3). However, allele-specific expression analysis revealed reactivation of the paternal MEG allele in only two out of eleven tested MEGs (*At5g03020*, *At2g19400*; Figure 4 and Figure 5), indicating that increased expression of MEGs in *fis2* mutants is not necessarily a consequence of paternal MEG allele activation, but is likely caused by an increased expression of the maternal MEG alleles.

Previous studies revealed global deregulation of FIS PcG target genes in response to interploidy crosses (2n × 4n) [46], [47]. Global deregulation of imprinted genes was furthermore proposed to account for interploidy seed defects [48]. Therefore, if the FIS PcG complex plays a major role in the regulation of imprinted genes, imprinted genes should become largely deregulated in response to interploidy crosses. We tested this hypothesis by analyzing MEG and PEG expression in seeds derived after pollination of diploid plants with tetraploid pollen donors. Indeed, most MEGs and PEGs that were deregulated in *fis2* were as well significantly deregulated in triploid seeds derived from interploidy crosses, adding support to this hypothesis (Figure 4, Figure 5, Figure 6, Figure S3 and Figure S4).

Together, our data reveal that maternal and paternal alleles of a subset of MEGs and maternal alleles of PEGs are regulated by the FIS PcG complex. The FIS PcG complex confers tight repression of the maternal PEG alleles and some paternal MEG alleles, whereas it mainly modulates expression of many maternal MEG alleles.

### MEGs and PEGs Are Often Neighboured by Transposable Elements

**Figure 7:**
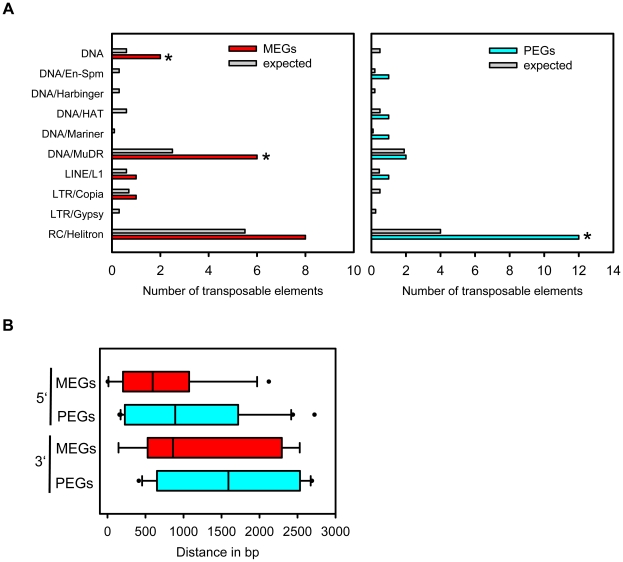
Types of Transposable Elements in the Vicinity of MEGs and PEGs. (A) Type of transposable elements present in MEGs (left panel) and PEGs (right panel) in comparison to their representation among detectable genes in our dataset (gray bars). TE superfamilies are as defined by TAIR (www.arabidopsis.org). (B) Distance of transposable elements in relation to the transcriptional start (5′ location) or stop (3′ location) of MEGs and PEGs.

Transposable elements have been implicated as the driving force for the evolution of imprinted gene expression [6], [7], [20]. Therefore, we addressed the question whether MEGs and PEGs have an increased likelihood to contain transposable elements in their vicinity compared to other detectable genes. Indeed, this test revealed a significantly increased likelihood for MEGs (p<0.009) as well as PEGs (p<1.3E-6). We also tested whether a particular subclass of transposable elements is enriched in the vicinity of MEGs and PEGs. Among the tested elements we noted a significant enrichment for RC/helitrons in PEGs (p<2.7 E-5). MEGs also had more RC/helitrons than expected by chance (8 versus 5), which was, however, not statistically significant (p<0.08). The presence of helitrons was previously reported within the 5′ region of imprinted genes *MEA* [49], *FWA* [31], *HDG3* and *HDG9* [6], implicating a functional association between the presence of helitrons and imprinted gene expression. Among MEGs we also noted a significant enrichment for MuDR (p = 0.01) and DNA transposable elements (p = 0.024), however, it is possible that (due to the relatively small sample size) enrichments for other elements were not detected. Correlating with a different CG DNA methylation pattern of MEGs and PEGs, the location of transposable elements in relation to the transcriptional start or stop differed between MEGs and PEGs (Figure 7B). PEGs had a much greater median distance of transposable elements in relation to the transcriptional start and stop compared to MEGs (Figure 7B), supporting previous observations of a distally located helitron remnant being required for imprinted expression of the PEG *PHE1* [19].

### MEGs and PEGs Are Faster Evolving When Compared to the Rest of the Genes in the *Arabidopsis thaliana* Genome

A number of studies of imprinted genes in both mammals and plants have found evidence that imprinted genes are rapidly evolving under positive Darwinian selection [27]–[29]. To determine whether either MEGs or PEGs displayed any evidence of fast evolution, pairwise *d*N/*d*S calculations were performed for the entire genome of *Arabidopsis thaliana*. Using reciprocal BLASTP searches, 19,965 orthologous pairs of proteins (gene models) were identified between *Arabidopsis thaliana* and *Arabidopsis lyrata* from the 27,235 *Arabidopsis thaliana* gene models tested. These included 27 (69.23%) of the MEGs and accession-dependent MEGs and 19 (70.37%) of the PEGs and accession-dependent PEGs identified in this study. Gene models not returning reciprocal BLAST results were not considered further for *d_N_*/*d_S_* analysis. Those gene models tested were then split into MEGs, PEGs and a third group representing the remaining genes tested. All three classes were then compared to determine whether there was any difference in relation to *d_N_*/*d_S_* values which measure rate of evolution of a protein coding-locus (Table S9).

In all three classes most genes have a *d_N_*/*d_S_* of less than one suggesting some level of purifying selection. However, the *d_N_*/*d_S_* value (as calculated here) is an average across the whole CDS sequence and masks potential heterogeneity of selection pressures across the gene. Despite this caveat, the *d_N_*/*d_S_* differences between imprinted and biallelically expressed genes is quite striking. For both MEGs and PEGs the reported median *d_N_*/*d_S_* is significantly higher than that of the background *d_N_*/*d_S_* for the remainder of the genome (p = 1.184e-05 and p = 2.991e-08 respectively, Wilcoxon Rank Sum), indicating that uniparentally expressed imprinted genes are fast evolving when compared to biallelically expressed genes (as represented by the background *d_N_*/*d_S_* values). However, although the median *d_N_*/*d_S_* for the MEGs is observed to be a third higher than that of the PEGs this difference is not reported as significant (p = 0.1614, Wilcoxon Rank Sum).

Also notable within the MEGs is that eight (∼30%) of all the MEGs tested (i.e. *At1g61090, At3g57250, At1g51000, At5g46300, At4g29570 At1g12180, At1g52460* and *At1g07690*) reported a *d_N_*/*d_S_* value greater than one providing particularly strong evidence of fast evolution for these genes.

Within the PEGs, only one of the genes (∼5%; *At2g20160*) displayed a *d_N_*/*d_S_* value greater than one. However, statistical testing did not reveal a significant difference between the number of fast evolving MEGs and PEGs (p = 0.0851, Fisher2019s exact test). Full details of the *d_N_*/*d_S_* analysis for both MEGs and PEGs is presented in Tables S10 and S11.

### A Subset of MEGs and PEGs Are Located in Clusters

**Figure 8:**
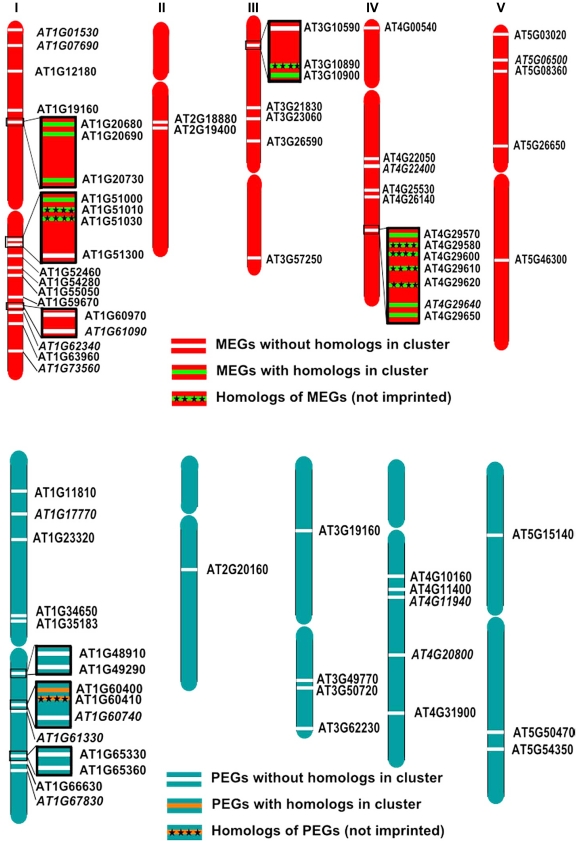
Some MEGs and PEGs Are Located in Clusters. Chromosomal distribution of MEGs and accession-dependent MEGs (A) and PEGs and accession-dependent PEGs (B) along the chromosomes. Accession-dependent MEGs and PEGs are italicized. Genes located in clusters are boxed. Clustered MEGs and PEGs having homologs within the cluster are highlighted in green and orange, respectively. Non-imprinted homologs of clustered MEGs and PEGs are indicated by cross signs.

Imprinted loci in mammals are clustered over megabase regions in the genome and this clustering is essential to their imprinted regulation [50]; raising the question whether imprinted loci in Arabidopsis are located within clusters as well. We searched for clustered MEGs and PEGs (including accession-dependent MEGs and PEGs) by applying a sliding window analysis. Using window sizes of 50 genes, significantly higher numbers of MEGs and PEGs were found to occur in clusters than expected by chance (p<0.05; Figure S7). We identified five MEG clusters containing two to three genes as well as three PEG clusters containing two genes (Figure 8A, 8B). Interestingly, most clusters contained either homologous MEGs or PEGs, or non-imprinted homologs of MEGs and PEGs (Figure 8A, 8B), implicating local sequence duplications as a driving force for the formation of imprinted genes. If so, there should be a higher incidence of imprinted genes having close sequence homologs compared to the genome-wide frequency of homologous genes. We tested this hypothesis by analyzing the number of close homologs to MEGs and PEGs and found indeed that MEGs and PEGs have an increased frequency of close homologs in comparison to the genome-wide frequency (p<0.05, Table S12), suggesting that gene duplications are in most cases connected with the formation of imprinted genes in Arabidopsis. Therefore, it seems possible that cluster formation of MEGs and PEGs is a consequence of local gene duplication and not essential for imprinted gene regulation, in agreement with the finding that only a subset of imprinted genes is localized in clusters.

## Discussion

Unravelling the biological significance of genomic imprinting is crucially dependent on the identification of the majority of imprinted gene loci. In our study we succeeded in identifying more than 60 potentially imprinted loci, with a similar ratio of specifically maternally and paternally expressed imprinted genes. We successfully identified six out of twelve previously known imprinted genes, proving that our strategy can successfully identify novel imprinted genes. Six previously identified imprinted genes were not identified either because these genes are only weakly expressed at 4 DAP (*MEA* and *FIS2*) [46], they lack polymorphic sites between Col-0 and Bur-0 or because of accession-dependent imprinting. We only considered a gene to be imprinted if it had significantly deviating read numbers from the expected maternal to paternal ratio in both directions of the crosses. Those genes that passed this significance threshold were again tested for significant deviating read numbers when comparing the maternal and paternal Col-0 alleles versus the maternal and paternal Bur-0 alleles. Based on this comparison about 10% of the identified MEGs and 20% of the identified PEGs are likely to be imprinted only in one accession, however, many accession-dependent imprinted genes did not pass our first significance threshold (e.g. *HDG3*, *HDG8*, *HDG9*), suggesting that the number of accession-dependent imprinted genes is significantly higher. It has been noted that there is a difference between Arabidopsis accessions in their tolerance to interploidy crosses [51], whether accession-dependent imprinted genes are the underlying cause for this phenomenon is an attractive hypothesis.

Using publicly available microarray datasets we stringently filtered our MEG dataset for genes that are not expressed in vegetative tissues and the seed coat. This filtering allowed us to predict genes with allele-specific expression in the endosperm, but using this strategy we lost genes that are either not present on the ATH1 microarray (about 25%) or that are expressed in vegetative tissues but are still regulated by genomic imprinting. However, the vast majority of known imprinted genes are not significantly expressed during vegetative development, suggesting that we have identified a significant number of MEGs present in the genome. Although PEGs were not filtered against vegetative or seed coat expression, the majority of PEGs were similarly excluded from expression in vegetative tissues, indicating that imprinted genes in Arabidopsis have mainly endosperm-restricted functions.

### Different Experimental Strategies Result in the Identification of Complementary Sets of Imprinted Genes

We compared the MEGs and PEGs identified in our study with MEGs and PEGs identified by a similar approach using the accession combinations Landsberg *erecta* (L*er*) and Col [45]. Whereas the majority of unfiltered MEGs identified by Hsieh and colleagues were present in our unfiltered MEG dataset (84%; 549 genes; Tables S13 and S14), only six genes were commonly identified as MEGs after filtering (Table S17; Figure S8A). The majority of MEGs defined by Hsieh and colleagues as being expressed in the endosperm were also present in our unfiltered MEG dataset (78%, 89 genes, Figure S8A; Table S15). However, when analyzing the expression of these genes within different seed tissues we found the majority of them being strongly expressed in the seed coat (Figure S9) and did, therefore, not pass our stringent filtering criteria. Only eight out of 39 predicted MEGs identified in this study were as well present in the unfiltered dataset of Hsieh and colleagues (Figure S8A; Table S16) [45], indicating that differences in the filtering cannot sufficiently explain the differences between the identified MEG datasets. Similarly, only seven out of 119 predicted unfiltered PEGs overlapped with the unfiltered PEG dataset of Hsieh and colleagues (Table S18 and Table S19, Figure S8B), supporting the view that differences in filtering strategies do not fully account for the different datasets. It thus seems likely that the different experimental setup between this study and the study by Hsieh and colleagues, including different accession combinations and different developmental stages (4 DAP versus 7–9 DAP in [45]) resulted in the identification of complementary datasets.

### Regulation of MEGs and PEGs by DNA Methylation and the FIS PcG Complex

Most PEGs were devoid of CG DNA methylation around the transcriptional start site, indicating that DNA methylation is not responsible for silencing of maternal PEG alleles. Instead, we provide evidence that silencing of at least some maternal PEG alleles is mediated by the FIS PcG complex. We found that PEG loci have high H3K27me3 levels in the endosperm, and importantly, many PEGs were activated upon loss of FIS function, which is likely contributed by reactivation of maternal PEG alleles. We also detected increased expression of MEGs upon loss of FIS function. However, this was not a necessary consequence of paternal MEG allele reactivation, but often caused by an activation of maternal MEG alleles, suggesting that endosperm hypomethylation makes maternal MEG alleles vulnerable to FIS silencing. This hypothesis is supported by previous findings from our group showing that genes and transposable elements are targeted by the FIS PcG complex in the endosperm, which are protected from PcG targeting by DNA methylation in vegetative tissues [52]. Which mechanism prevents the FIS complex from targeting paternal alleles of PEGs? We previously showed that DNA demethylation of a distally located region together with the promoter-localized FIS PcG complex are required for silencing of maternal *PHE1* alleles, suggesting that long-range interactions of sequence elements are required for efficient silencing of maternal *PHE1* alleles [19]. Here, we show that PEGs are flanked by regions of high CG DNA methylation levels, suggesting that upon DNA demethylation in the endosperm these regions are targeted by the FIS PcG complex, conferring tight repression of maternal PEG alleles. In support of this hypothesis, we found high H3K27me3 levels in PEG flanking regions, which were much higher than the H3K27me3 levels present in vegetative tissues (Figure 6, Figure S4 and Figure S6).

The impact of hypomethylation on the activity status of the paternal MEG alleles was contrasted by the lack of CG DNA methylation in the immediate vicinity of several MEGs. However, MEGs lacking CG methylation often contained substantial levels of non-CG DNA methylation (Figure S5), implicating that non-CG DNA methylation regulates paternal MEG alleles. Reactivation of these alleles upon loss of MET1 function might be a consequence of a proposed feedback between CG and non-CG DNA methylation [53], [54]. Therefore, it seems possible that the paternal alleles of some MEGs are silenced by non-CG methylation established specifically in the endosperm and reactivation of the paternal alleles requires loss of non-CG methylation.

In stark contrast to silencing of MEGs and PEGs during vegetative development, many MEGs and PEGs were expressed in pollen. The vegetative cell of pollen has low levels of DNA methylation [55], suggesting that reduced levels of DNA methylation in the vegetative cell cause activation of MEGs, similar to the activation of maternal MEG alleles in the endosperm. Whether activation of PEGs in pollen is caused by reduced PcG protein activity in the vegetative cell of pollen remains to be tested.

### Evolution of Imprinted Genes

Our study also sheds light on the evolution of imprinted genes, as we found a significant enrichment of transposon insertions in vicinity to MEGs and PEGs. In particular helitrons were strongly enriched in the vicinity of PEGs and were also overrepresented (albeit not statistically significant) in the vicinity of MEGs. Helitrons are eukaryotic DNA transposons which constitute >2% of the Arabidopsis genome. A striking feature of helitrons is their capacity to capture and propagate host genes, making them powerful factors shaping the evolution of genomes [56]. Although the rate of gene capture in Arabidopsis is predicted be to low compared to the major occurrence of gene capture in maize [57], it is possible that these predictions are a drastic underestimation due to rapid purging of helitron elements in Arabidopsis [58]. Thus, it is possible that helitron-mediated gene duplications which generate increased gene dosage may set the ground for imprinted gene evolution. Interestingly, we found a higher incidence of MEGs and PEGs having close sequence homologs, however, whether these duplications are a consequence of helitron activity remains to be shown.

If parental conflicts involving imprinted genes are mediated by amino-acid changes in the gene products of imprinted loci under antagonistic co-evolution, such imprinted loci may be subject to rapid evolution, possibly under positive Darwinian selection [27]–[29]. The identification of 60 potentially imprinted genes in Arabidopsis provided the opportunity for an initial exploration of whether the MEGs and PEGs identified displayed any evidence of rapid evolution. When the *d_N_*/*d_S_* values of the imprinted genes (MEGs and PEGs and accession-dependent MEGs and PEGs) were compared with the average *d_N_*/*d_S_* values for all other genes in the genome it is clear that imprinted genes in Arabidopsis are more rapidly evolving. Furthermore, a significant proportion of the MEGs displayed *d_N_*/*d_S_* values greater than 1 which is indicative of fast evolving genes. Further sequencing of these imprinted genes in populations and outgroup species will determine whether these genes are undergoing positive Darwinian selection or are under relaxed constraints.

Imprinted genes are predominantly expressed in the endosperm, implicating a specific role of these genes during endosperm development. Alternatively, it is possible that imprinted genes are on the trajectory to become pseudogenes and therefore, are silenced during vegetative development. However, the fact that many imprinted genes have functional roles as transcription factors or have chromatin modifying activity supports a proposed functional role of imprinted genes for endosperm development. To identify these functions and to test whether MEGs and PEGs have indeed antagonistic roles in controlling endosperm growth as it has been proposed previously [21], [22], will remain the challenge of future investigations.

## Materials and Methods

### Plant Material and Growth Conditions

The *Arabidopsis thaliana* accessions used in this study were Col-0 and Bur-0. The *fis2-5* and *met1-3* mutants (both in Col accession) were described previously [52], . The newly identified *fie-12* allele (GK_362D08; Col-0 accession) contains a T-DNA insertion within the third exon. The *fie-12* seed abortion ratio and mutant seed phenotypes were analyzed and found to be similar to previously published *fie* alleles (data not shown). All mutants were heterozygous and the genotype confirmed by PCR analysis. Plants were grown in a growth cabinet under long day photoperiods (16 h light and 8 h dark) at 22°C. After 10 days, seedlings were transferred to soil and plants were grown in a growth chamber at 60% humidity and daily cycles of 16 h light at 22°C and 8 h darkness at 18°C. For reciprocal crosses, designated female partners were emasculated, and the pistils hand-pollinated two days after emasculation.

### Phenotypic Analysis of Seed Development in Col-0 and Bur-0 Accessions

To control for variations in seed development between Col-0 and Bur-0 accessions, siliques were harvested 4 DAP and fixed overnight in 9:1 ethanol:acetic acid. Siliques were dissected to release seeds into clearing solution (67% chloralhydrate in 8% glycerol) for overnight incubation. Microscopy imaging was performed using a Leica DM 2500 microscope using DIC optics (Leica Microsystems, Wetzlar, Germany), images were captured using a Leica DFC300 FX digital camera (Leica) and exported using Leica Application Suite Version 2.4.0.R1 (Leica Microsystems) and processed using Photoshop 7.0 (Adobe).

### RNA Extraction and cDNA Synthesis

Seeds of at least 40 siliques per sample were harvested into 50 µl RNAlater (Sigma, Buchs, Switzerland) at 4 DAP. Glass beads (1.25–1.55 mm) were added, and the samples were ground unfrozen in a Silamat S5 (Ivoclar Vivadent, Ellwangen, Germany). RNA was extracted using the RNeasy Plant Mini Kit (Qiagen, Hilden, Germany) according to the manufacturer's instructions. For cDNA synthesis residual DNA was removed using the Qiagen RNase-free DNase Set and cDNA was synthesized using the Fermentas First strand cDNA synthesis kit (Fermentas, Burlington, Canada) according to the manufactureŕs instruction.

### Preparation of mRNA-Sequencing Libraries

Sequencing libraries were prepared with the Illumina mRNA-Seq Sample Prep Kit (Illumina, San Diego, USA) according to the manufacturer's instructions. After adapter ligation library fragments of ∼250 bp were isolated from an agarose gel. The DNA was PCR amplified with Illumina primers for 15 cycles, purified and loaded on an Illumina flow cell for cluster generation. Libraries were sequenced on the Illumina Genome Analyzer II following the manufacturer's protocols.

### Bioinformatic Analysis

#### Identification of SNP-associated reads

TAIR8 chromosome sequences were downloaded from TAIR (ftp.arabidopsis.org/home/tair/Genes/TAIR8_genome_release/tair8.at.chromosomes.fas, chromosomes 1 to5), together with the TAIR8 genome annotation (ftp.arabidopsis.org/home/tair/Genes/TAIR8_genome_release/TAIR8_gff3/TAIR8_GFF3_genes.gff). Bur-0 SNPs [30], 569,859 SNPs in total for chromosomes 1 to 5, were downloaded from the 1001 genomes website (http://1001genomes.org/data/MPI/MPIOssowski2008/releases/2008_06_05/strains/Bur-0/Bur-0_homozygous_snp_080605.txt). For each of the 569,859 Col-0/Bur-0 SNPs, we extracted a 71nt genomic window around the SNP (SNP position plus/minus 35nt) from the Col-0 reference sequence and annotated it as a Col-0 window. The nucleotide in the SNP was then replaced by the Bur-0 variant and the resulting sequence annotated as a Bur-0 window.

A suffix array was constructed from the union of Col-0 and Bur-0 windows with the mkvtree program (http://www.vmatch.de/) [60]. RNAseq reads were mapped with the vmatch program (http://www.vmatch.de/) in both forward and reverse complementary orientation (options -d and -p), allowing up to two mismatches (option -h 2), requiring the whole read to map (option -l 36), and generating maximal substring matches that are unique in the Col-0/Bur-0 window dataset (option -mum cand). This procedure resulted in 8,576,779 matches for Bur-x-Col reads (out of 102,705,076 total reads) and 6,773,239 matches for Col-x-Bur reads (out of 122,367,092 total reads). SNP windows were associated with gene ids via the TAIR8 genome annotation by overlapping with or being included in a gene region (gene start to end, ignoring exon/intron structure). SNP windows that were associated with more than one gene were discarded. From the remaining SNP windows, the grand total associated with a gene was defined as that gene's expression level. 147,349 SNP windows were matched by at least one read in one of the two reciprocal experiments, accounting for 19,161 genes, out of 25,189 genes that had at least one SNP in at least one exon, out of 28,523 annotated genes in total. Sequencing raw data generated in this study are available at GEO, accession number GSE27292.

#### Testing for allele-specific expression

For each gene and cross (Col-0×Bur-0 and Bur-0×Col-0), we performed a binomial one-sided test against the null-hypothesis of 2m∶1p expression. The resulting p-values were the probabilities of deviation from the expected 2m∶1p ratio towards either larger maternal expression or larger paternal expression under the null-hypothesis of an unbiased 2m∶1p expression. The two p-values for maternal expression from the two reciprocal crosses (p1, p2) were summarized in a joint p-value based on the distribution of the second-order statistic by calculating p = max(p1,p2)∧2. Joint p-values for paternal expression were calculated analogously. Joint p-values, either describing reciprocal maternal expression or reciprocal paternal expression, were sorted in ascending order (from significant to insignificant), and for each joint p-value the false-discovery rate (FDR) [61] was calculated, as q = p*n/i, where n was the overall number of joint p-values and i was the rank of a given p-value. Genes with a q-value of 0.05 or less were selected as maternally or paternally expressed genes.

Parental-specific splicing was tested by analyzing for every candidate gene the numbers of reads across all SNPs of that gene, using Pearson's chi-square test (R function chisq.test with parameter simulate.p.value = T).

#### Filtering for endosperm-expressed genes and analysis of MEGs and PEGs

Filtering for endosperm-specific gene expression was performed by using data from endosperm transcript profiles generated in the laboratories of Bob Goldberg (UCLA), John Harada (UC Davis), Brandon Le (UCLA), Anhthu Bui (UCLA), and Julie Pelletier (UC Davis) that are available under http://estdb.biology.ucla.edu/genechip/project [62]. The same data were used to generate Figure 3. Reference transcript profiles from vegetative tissues (seedlings, cotyledons, hypocotyl, leaves, stems, roots, shoot apical meristem), flowers and siliques were published by [63]. Genes were considered as preferentially expressed in the endosperm if the SLRs in one of the endosperm domains were at least fivefold higher than the SLRs of the seed coat and SLRs were below five in vegetative tissues. MEGs with low mRNA levels (read counts higher or equal 10 and smaller or equal 30) were considered as being endosperm-preferred expressed if SLRs in one of the endosperm domains were at least threefold higher compared to the seed coat and SLRs were below five in vegetative tissues (Table S3). Genes were considered as being expressed in the endosperm with SLRs>4.5 in at least one of the endosperm domains (Table S5). The transcript profiles of wild-type and *fis2* seeds at 3 and 6 DAP as well as seeds derived from interploidy crosses were published by [46], [52]. H3K27me3 profiling data from vegetative tissues and the endosperm were published by [52], [64]. Clustering analysis of expression profiles was performed using TM4 software [65]. DNA methylation profiles were taken from [7], [41] and were visualized using R software (http://www.r-project.org/). Enrichment of GO categories (obtained from TAIR) was tested based on the hypergeometric test and multiple-testing correction according to [61] with a critical p-value of 5.0E-03. Homologous genes were identified using the blastp program from blastall (http://www.ncbi.nlm.nih.gov/staff/tao/URLAPI/blastall/) by applying matrix BLOSUM62 and a critical e-value of 0.01. Identification of MEG and PEG clusters was performed by establishing the frequency of MEGs and PEGs present in windows of a defined size using a sliding window analysis. p values were calculated from a reference distribution that was based on an identical number of randomly sampled genes. Transposable elements were identified based on information in the TAIR database (ftp://ftp.arabidopsis.org/home/tair/Genes/TAIR8_genome_release/TAIR8_Transposable_Elements.txt). Statistical testing was performed using a hypergeometric test as well as a permutation test. Both tests gave the same result.

#### Testing for evidence of rapid evolution of MEGs and PEGs

Reciprocal BLASTP searches were performed between *Arabidopsis thaliana* versus *Arabidopsis lyrata* (i.e. *Arabidopsis thaliana* peptides versus *Arabidopsis lyrata* peptide database and *Arabidopsis lyrata* peptides versus *Arabidopsis thaliana* peptide database) of all MEGs and PEGs listed in Tables S4 and S7, respectively. The reciprocal top hit sequences were then aligned at the peptide level using MUSCLE [66]. Using the peptide alignment as a template the reciprocal top hit CDS sequences were then aligned using the tranalign [67]. Pairwise *d_N_*/*d_S_* analysis was then performed on the CDS alignments using both the CodeML program (using model 0 and runmode -2) and yn00 both from the PAML package [68]. The values from CodeML are reported in the main text, values for both CodeML and yn00 are found in Tables S9, S10 and S11.

### Validation of RNA-Sequencing Results

Selected loci were validated using independently prepared RNAs from reciprocal crosses between Col-0 and Bur-0. Primers used for allele specific expression analysis of selected genes are specified in Table S20. The amplified products were either digested with indicated restriction enzymes (Table S20) and analyzed on agarose gels or by DNA sequencing.

## Supporting Information

Figure S1 Expression of PEGs in Vegetative and Seed Tissues. (A) Cluster analysis of PEGs and accession-dependent PEGs based on their expression in vegetative tissues and seeds. PEGs were grouped into two mutually exclusive clusters based on their expression in pollen. The cluster containing genes with low or without expression in pollen is marked by a light blue bar; the cluster containing genes with high pollen expression is marked with a dark blue bar. Each row represents a gene, and each column represents a tissue type. Tissue types are: seedlings, cotyledons, hypocotyl, leaves, stems, roots, shoot apical meristem (SAM), flowers at stages 10, 12, 15, siliques containing seeds with embryos in the globular to heart stage, heart stage and torpedo stage. Red or green indicate tissues in which a particular gene is highly expressed or repressed, respectively. (B) Cluster analysis of PEGs and accession-dependent PEGs based on their expression in embryo, endosperm and seed coat during different stages of seed development. PEGs were grouped into three mutually exclusive clusters based on their expression in embryo and the endosperm. The cluster containing genes with low or without expression in embryo and endosperm is marked with a light orange bar, the cluster containing genes with low expression in the embryo but high endosperm expression is marked with a dark orange bar, and the cluster containing genes with high expression in embryo but low or without expression in the endosperm is marked with a red bar. Each row represents a gene, and each column represents a tissue type. Tissue types are: embryos from the preglobular stage to the mature stage, micropylar (MPE), peripheral (PE) and chalazal (CZE) endosperm derived from seeds containing embryos of the preglobular stage to the mature stage, and seed coat derived from seeds containing embryos of the preglobular stage to the mature stage. Red or green indicate tissues in which a particular gene is highly expressed or repressed, respectively.
(PDF)

Figure S2 Interaction Network of AGL Transcription Factors Based on Yeast Two Hybrid Interaction Data [37]. Maternally expressed MEGs are indicated in red, paternally expressed AGLs are indicated in blue. The central regulator AGL62 is depicted in orange.
(PDF)

Figure S3 CG DNA Methylation Profiles of MEGs in Vegetative Tissues and Endosperm and Expression Analysis of MEGs in *fis2* and 2n×4n Interploidy Crosses. (A–C) Analysis of MEGs without prominent CG DNA methylation levels in the vicinity of genic regions. (D–F) Analysis of MEGs with prominent CG DNA methylation levels in the vicinity of genic regions. (A, D) Fold-changes of MEG expression in *fis2* mutant seeds at 3 and 6 days after pollination (DAP) and from seeds derived from pollination with tetraploid pollen donors at 6 DAP compared to wild-type seeds at the corresponding time points. Data are based on ATH1 microarray signals after RMA normalization. Significantly deregulated genes are marked by an asterisk. (B, E) CG DNA methylation profiles of indicated MEGs in vegetative tissues (black line) or endosperm (red line) based on data published by [7], [41]. The gray bar represents the annotated gene body from transcription start (left) to transcription end (right). Red boxes represent transposable elements. Profiles are shown for 5% length intervals along the gene body and for 100 bp sequence intervals for the 2-kb regions upstream and downstream of each gene. The vertical dotted lines mark the gene body. The horizontal dashed line marks the DNA methylation level in vegetative tissues of TAIR8-annotated genes at the transcriptional start site. (C, F) H3K27me3 profiles of indicated MEGs in vegetative tissues (black line) or endosperm (red line) based on data published by [52], [64]. The gray bar represents the annotated gene body from transcription start (left) to transcription end (right). Red boxes represent transposable elements. Profiles are shown for 5% length intervals along the gene body and for 100 bp sequence intervals for the 2-kb regions upstream and downstream of each gene. The vertical dotted lines mark the gene body. The horizontal dashed line marks the H3K27me3 level of TAIR8-annotated genes at the transcriptional start site.
(PDF)

Figure S4 CG DNA Methylation Profiles of PEGs in Vegetative Tissues and Endosperm and Expression Analysis of PEGs in *fis2* and 2n×4n Interploidy Crosses. (A) Fold-changes of PEG expression in *fis2* mutant seeds at 3 and 6 days after pollination (DAP) and from seeds derived from pollination with tetraploid pollen donors at 6 DAP compared to wild-type seeds at the corresponding time points. Data are based on ATH1 microarray signals after RMA normalization. Significantly deregulated genes are marked by an asterisk. (B) CG DNA methylation profiles of indicated PEGs in vegetative tissues (black line) or endosperm (red line) based on data published by [7], [41]. The gray bar represents the annotated gene body from transcription start (left) to transcription end (right). Red boxes represent transposable elements. Profiles are shown for 5% length intervals along the gene body and for 100 bp sequence intervals for the 2-kb regions upstream and downstream of each gene. The vertical dotted lines mark the gene body. The horizontal dashed line marks the DNA methylation level in vegetative tissues of TAIR8-annotated genes at the transcriptional start site. (C) H3K27me3 profiles of indicated PEGs in vegetative tissues (black line) or endosperm (red line) based on data published by [52], [64]. The gray bar represents the annotated gene body from transcription start (left) to transcription end (right). Red boxes represent transposable elements. Profiles are shown for 5% length intervals along the gene body and for 100 bp sequence intervals for the 2-kb regions upstream and downstream of each gene. The vertical dotted lines mark the gene body. The horizontal dashed line marks the H3K27me3 level of TAIR8-annotated genes at the transcriptional start site.
(PDF)

Figure S5 CHG and CHH Methylation Profiles of MEGs. (A, B) CHG (A) and CHH (B) DNA methylation profiles of MEGs shown in Figure 4 in vegetative tissues (black line) or endosperm (red line) based on data published by [7], [41]. (C, D) CHG (C) and CHH (D) DNA methylation profiles of MEGs shown in Figure S3A in vegetative tissues (black line) or endosperm (red line) based on data published by [7], [41]. The gray bar represents the annotated gene body from transcription start (left) to transcription end (right). Red boxes represent transposable elements. Profiles are shown for 5% length intervals along the gene body and for 100 bp sequence intervals for the 2-kb regions upstream and downstream of each gene. The vertical dotted lines mark the gene body. The horizontal dashed line marks the DNA methylation level in vegetative tissues of TAIR8-annotated genes at the transcriptional start site.
(PDF)

Figure S6 Average H3K27me3 profiles of vegetative tissues and endosperm. Average H3K27me3 profiles of vegetative tissues (black line) or endosperm (red line) of TAIR8-annotated genes (left panels), MEGs (middle panels), and PEGs (right panels). MEGs and PEGs correspond to all genes indicated in Tables S4 and S7, respectively. The gray bar represents the annotated gene body from transcription start (left) to transcription end (right). Profiles are shown for 5% length intervals along the gene body and for 100 bp sequence intervals for the 2-kb regions upstream and downstream of each gene. The vertical dotted lines mark the gene body. The horizontal dashed line marks the H3K27me3 level of TAIR8-annotated genes at the transcriptional start site.
(PDF)

Figure S7 Identification of windows containing significantly enriched numbers of clustered MEGs (A) and PEGs (B). The significance threshold (p = 0.05) is indicated by a red line.
(PDF)

Figure S8 Overlap of MEGs (A) and PEGs (B) identified by [45] and MEGs and PEGs identified in this study. MEGs_LC and PEGs_LC correspond to MEGs and PEGs identified by [45] using L*er*/Col accessions; MEGs_BC and PEGs_BC correspond to MEGs and PEGs identified in this study using Bur-0/Col-0 accessions. Unfiltered MEGs_LC and PEGs_LC were identified by [45] using p≤0.001 and p≤0.05, respectively. Unfiltered MEGs_BC and PEGs_BC correspond to data shown in Tables S1 and S2, respectively. Filtered MEGs_BC and PEGs_BC correspond to data shown in Tables S4 and S7, respectively.
(PDF)

Figure S9 Cluster analysis of MEGs identified by [45] that overlap with unfiltered MEGs identified in this study (Table S15). Cluster analysis of MEGs was based on their expression in embryo, endosperm and seed coat during different stages of seed development. The cluster containing genes with low or without expression in seed coat is marked by a vertical orange bar. The cluster containing genes with high expression in the seed coat is marked by a vertical yellow bar. Genes present in our filtered MEG dataset (Table S7) are indicated. Each row represents a gene, and each column represents a tissue type. Tissue types are: embryos from the preglobular stage to the mature stage, micropylar (MPE), peripheral (PE) and chalazal (CZE) endosperm derived from seeds containing embryos of the preglobular stage to the mature stage, and seed coat derived from seeds containing embryos of the preglobular stage to the mature stage. Red or green indicate tissues in which a particular gene is highly expressed or repressed, respectively.
(PDF)

Table S1 Genes with maternally-biased expression (MEGs). m, Maternal, p, Paternal alleles.
(XLSX)

Table S2 Genes with paternally-biased expression (PEGs). m, Maternal, p, Paternal alleles.
(XLSX)

Table S3 Genes that are expressed in at least one stage and domain of endosperm development until heart stage and are not expressed in the seed coat and in vegetative tissues (seedlings, cotyledons, hypocotyl, leaves, stems, roots, shoot apical meristem, flowers at stages 10, 12).
(XLSX)

Table S4 MEGs and accession-dependent MEGs. m, Maternal, p, Paternal alleles. Genes marked in yellow are PcG target genes in vegetative tissues. Genes marked in orange have higher expression levels in flowers stage 12 than in siliques containing seeds with globular stage embryos (SLRs_Seeds_-SLRs_Flowers_). AD, accession-dependent imprinting; + imprinting confirmed; -, imprinting not confirmed.
(XLSX)

Table S5 Genes that are expressed in at least one stage and domain of endosperm development until heart stage.
(XLSX)

Table S6 Genes with paternally-biased expression (PEGs). m, Maternal, p, Paternal alleles.
(XLSX)

Table S7 PEGs and accession-dependent PEGs. m, Maternal, p, Paternal alleles. Genes marked in yellow are PcG target genes in vegetative tissues. AD, accession-dependent imprinting; + imprinting confirmed; -, imprinting not confirmed.
(XLSX)

Table S8 GO analysis of MEGs and PEGs (including accession-dependent MEGs and PEGs).
(PDF)

Table S9 Median omega, dN and dS values as calculated from pairwise alignments between *Arabidopsis thaliana* and *Arabidopsis lyrata* orthologs. Measure of spread represented as the semi interquartile range.
(PDF)

Table S10 Outputs for MEGs from Codeml and YN00 programs from the Codeml package.
(XLSX)

Table S11 Outputs for PEGs from Codeml and YN00 programs from the Codeml package.
(XLSX)

Table S12 Number of homologous genes to MEGs and PEGs (including accession-dependent MEGs and PEGs) in comparison to the genome-wide frequency of gene homologs.
(PDF)

Table S13 Comparative analysis of parent-of-origin specific genes identified in this study and published in [45]. Overlap of unfiltered MEGs_LC (p<0.05) and unfiltered MEGs_BC (Table S1). MEGs_LC, MEGs identified by Hsieh and colleagues [45] using L*er*/Col-0 accessions; MEGs_BC, MEGs identified in this study using Bur-0/Col-0 accessions.
(XLSX)

Table S14 Comparative analysis of parent-of-origin specific genes identified in this study and published in [45]. Overlap of unfiltered MEGs_LC (p<0.001) and unfiltered MEGs_BC (Table S1). MEGs_LC, MEGs identified by Hsieh and colleagues [45] using L*er*/Col-0 accessions; MEGs_BC, MEGs identified in this study using Bur-0/Col-0 accessions.
(XLSX)

Table S15 Comparative analysis of parent-of-origin specific genes identified in this study and published in [45]. Overlap of MEGs_LC (p<0.001 filtered for endosperm expression) and unfiltered MEGs_BC (Table S1). MEGs_LC, MEGs identified by Hsieh and colleagues [45] using L*er*/Col-0 accessions; MEGs_BC, MEGs identified in this study using Bur-0/Col-0 accessions.
(XLSX)

Table S16 Comparative analysis of parent-of-origin specific genes identified in this study and published in [45]. Overlap of unfiltered MEGs_LC (p<0.001) and filtered MEGs_BC (Table S7). MEGs_LC, MEGs identified by Hsieh and colleagues [45] using L*er*/Col-0 accessions; MEGs_BC, MEGs identified in this study using Bur-0/Col-0 accessions. Genes marked in yellow have been identified as accession-dependent MEGs_BC.
(XLSX)

Table S17 Comparative analysis of parent-of-origin specific genes identified in this study and published in [45]. Overlap of filtered MEGs_LC (p<0.001) and filtered MEGs_BC (Table S7). MEGs_LC, MEGs identified by Hsieh and colleagues [45] using L*er*/Col-0 accessions; MEGs_BC, MEGs identified in this study using Bur-0/Col-0 accessions. Genes marked in yellow have been identified as accession-dependent MEGs_BC.
(XLSX)

Table S18 Comparative analysis of parent-of-origin specific genes identified in this study and published in [45]. Overlap of unfiltered PEGs_LC (p<0.05) and unfiltered PEGs_BC (Table S2). PEGs_LC, PEGs identified by Hsieh and colleagues [45] using L*er*/Col-0 accessions; PEGs_BC, PEGs identified in this study using Bur-0/Col-0 accessions. Genes marked in red are not present on the ATH1 miroarray. Genes marked in yellow have been identified as accession-dependent PEGs_BC.
(XLSX)

Table S19 Comparative analysis of parent-of-origin specific genes identified in this study and published in [45]. Overlap of unfiltered PEGs_LC (p<0.05) and filtered PEGs_BC (Table S7). PEGs_LC, PEGs identified by Hsieh and colleagues [45] using L*er*/Col-0 accessions; PEGs_BC, PEGs identified in this study using Bur-0/Col-0 accessions. Genes marked in yellow have been identified as accession-dependent PEGs_BC.
(XLSX)

Table S20 Primers and enzymes used for allele-specific expression analysis.
(PDF)
