# Supplementary figures and images for: High-Resolution Analysis of Parent-of-Origin Allelic Expression in the Arabidopsis Endosperm
Source: PLoS Genet. 2011 Jun 16;7(6):e1002126. doi: 10.1371/journal.pgen.1002126 (PMC3116908; doi:10.1371/journal.pgen.1002126)

Figure S1

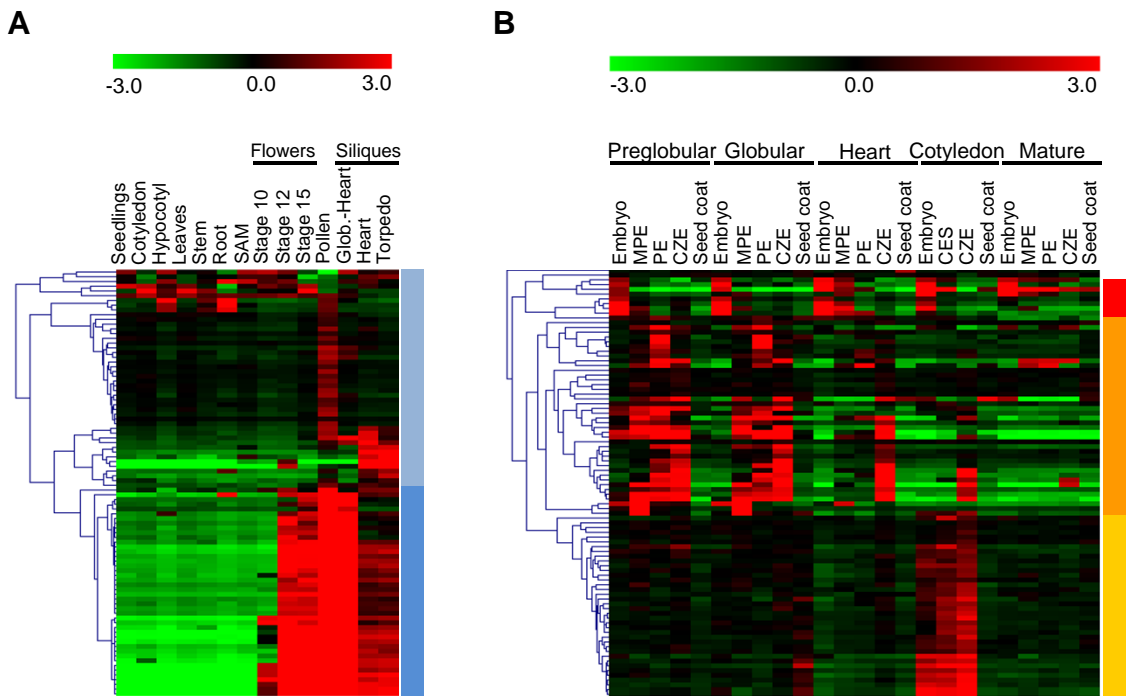

Supplement: Figure S1 — Expression of PEGs in Vegetative and Seed Tissues. (A) Cluster analysis of PEGs and accession-dependent PEGs based on their expression in vegetative tissues and seeds. PEGs were grouped into two mutually exclusive clusters based on their expression in pollen. The cluster containing genes with low or without expression in pollen is marked by a light blue bar; the cluster containing genes with high pollen expression is marked with a dark blue bar. Each row represents a gene, and each column represents a tissue type. Tissue types are: seedlings, cotyledons, hypocotyl, leaves, stems, roots, shoot apical meristem (SAM), flowers at stages 10, 12, 15, siliques containing seeds with embryos in the globular to heart stage, heart stage and torpedo stage. Red or green indicate tissues in which a particular gene is highly expressed or repressed, respectively. (B) Cluster analysis of PEGs and accession-dependent PEGs based on their expression in embryo, endosperm and seed coat during different stages of seed development. PEGs were grouped into three mutually exclusive clusters based on their expression in embryo and the endosperm. The cluster containing genes with low or without expression in embryo and endosperm is marked with a light orange bar, the cluster containing genes with low expression in the embryo but high endosperm expression is marked with a dark orange bar, and the cluster containing genes with high expression in embryo but low or without expression in the endosperm is marked with a red bar. Each row represents a gene, and each column represents a tissue type. Tissue types are: embryos from the preglobular stage to the mature stage, micropylar (MPE), peripheral (PE) and chalazal (CZE) endosperm derived from seeds containing embryos of the preglobular stage to the mature stage, and seed coat derived from seeds containing embryos of the preglobular stage to the mature stage. Red or green indicate tissues in which a particular gene is highly expressed or repressed, r [file pgen.1002126.s001.pdf]

Figure S2

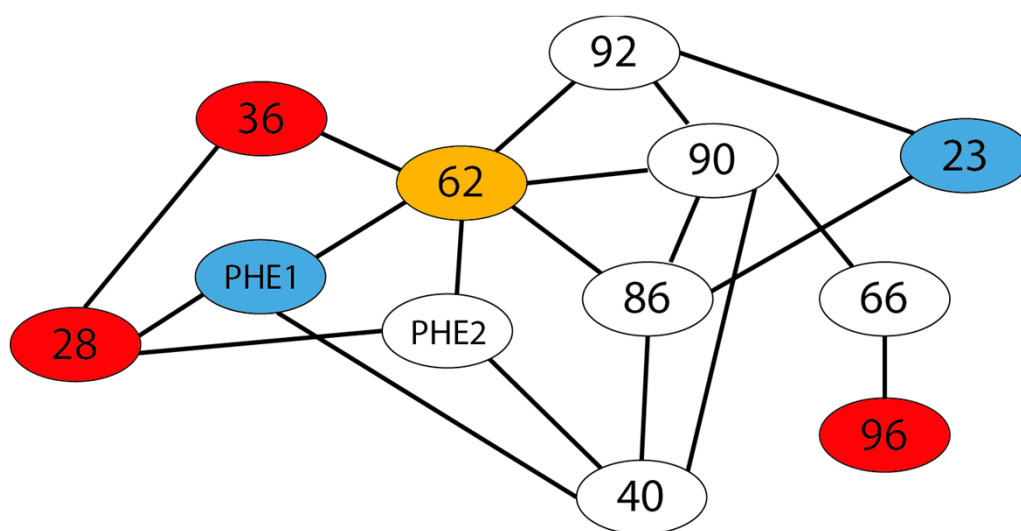

Supplement: Figure S2 — Interaction Network of AGL Transcription Factors Based on Yeast Two Hybrid Interaction Data [37]. Maternally expressed MEGs are indicated in red, paternally expressed AGLs are indicated in blue. The central regulator AGL62 is depicted in orange. (PDF) [file pgen.1002126.s002.pdf]

**Figure S3**

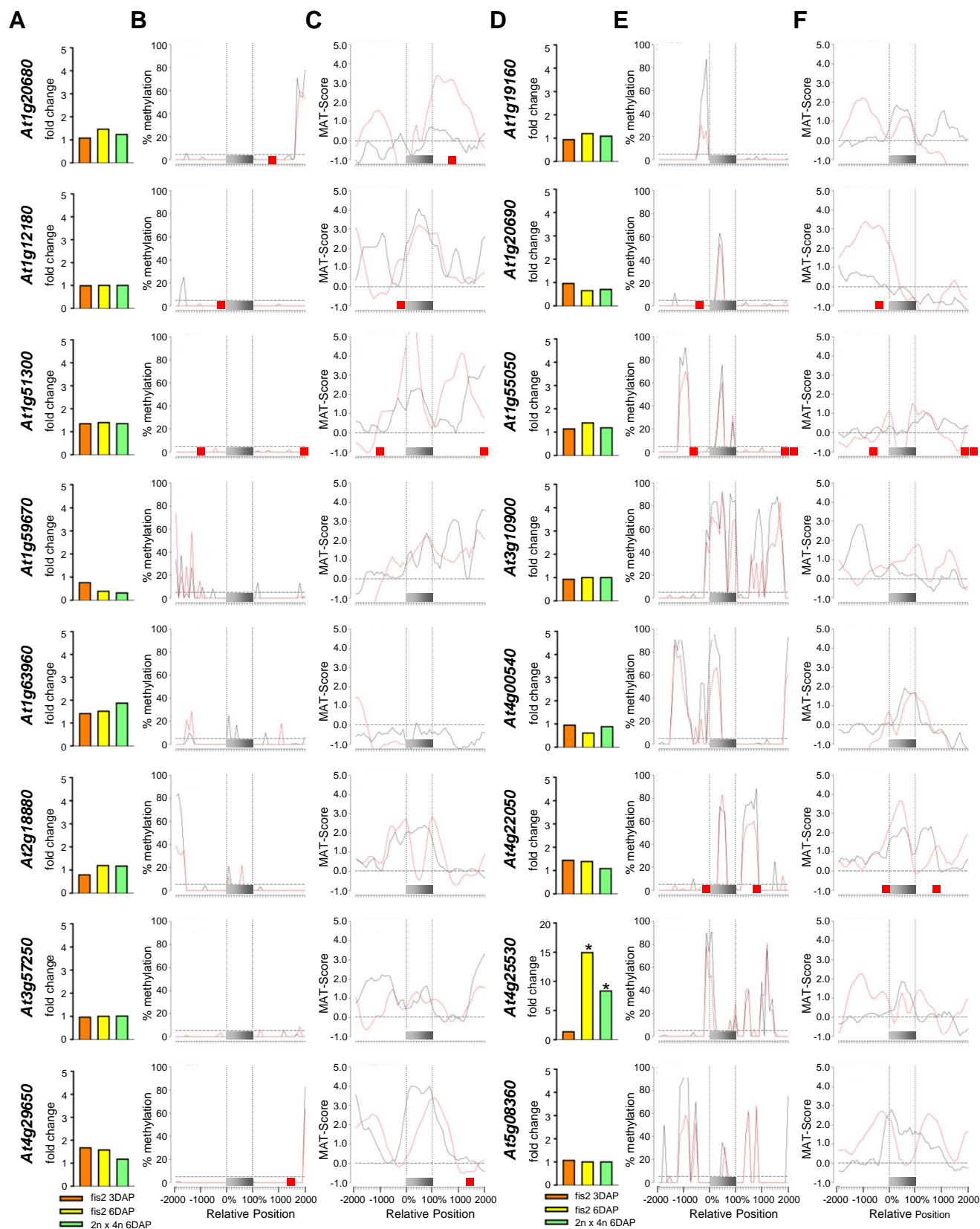

Supplement: Figure S3 — CG DNA Methylation Profiles of MEGs in Vegetative Tissues and Endosperm and Expression Analysis of MEGs in fis2 and 2n×4n Interploidy Crosses. (A–C) Analysis of MEGs without prominent CG DNA methylation levels in the vicinity of genic regions. (D–F) Analysis of MEGs with prominent CG DNA methylation levels in the vicinity of genic regions. (A, D) Fold-changes of MEG expression in fis2 mutant seeds at 3 and 6 days after pollination (DAP) and from seeds derived from pollination with tetraploid pollen donors at 6 DAP compared to wild-type seeds at the corresponding time points. Data are based on ATH1 microarray signals after RMA normalization. Significantly deregulated genes are marked by an asterisk. (B, E) CG DNA methylation profiles of indicated MEGs in vegetative tissues (black line) or endosperm (red line) based on data published by [7], [41]. The gray bar represents the annotated gene body from transcription start (left) to transcription end (right). Red boxes represent transposable elements. Profiles are shown for 5% length intervals along the gene body and for 100 bp sequence intervals for the 2-kb regions upstream and downstream of each gene. The vertical dotted lines mark the gene body. The horizontal dashed line marks the DNA methylation level in vegetative tissues of TAIR8-annotated genes at the transcriptional start site. (C, F) H3K27me3 profiles of indicated MEGs in vegetative tissues (black line) or endosperm (red line) based on data published by [52], [64]. The gray bar represents the annotated gene body from transcription start (left) to transcription end (right). Red boxes represent transposable elements. Profiles are shown for 5% length intervals along the gene body and for 100 bp sequence intervals for the 2-kb regions upstream and downstream of each gene. The vertical dotted lines mark the gene body. The horizontal dashed line marks the H3K27me3 level of TAIR8-annotated genes at the transcriptional start site. (PDF) [file pgen.1002126.s003.pdf]

**Figure S4**

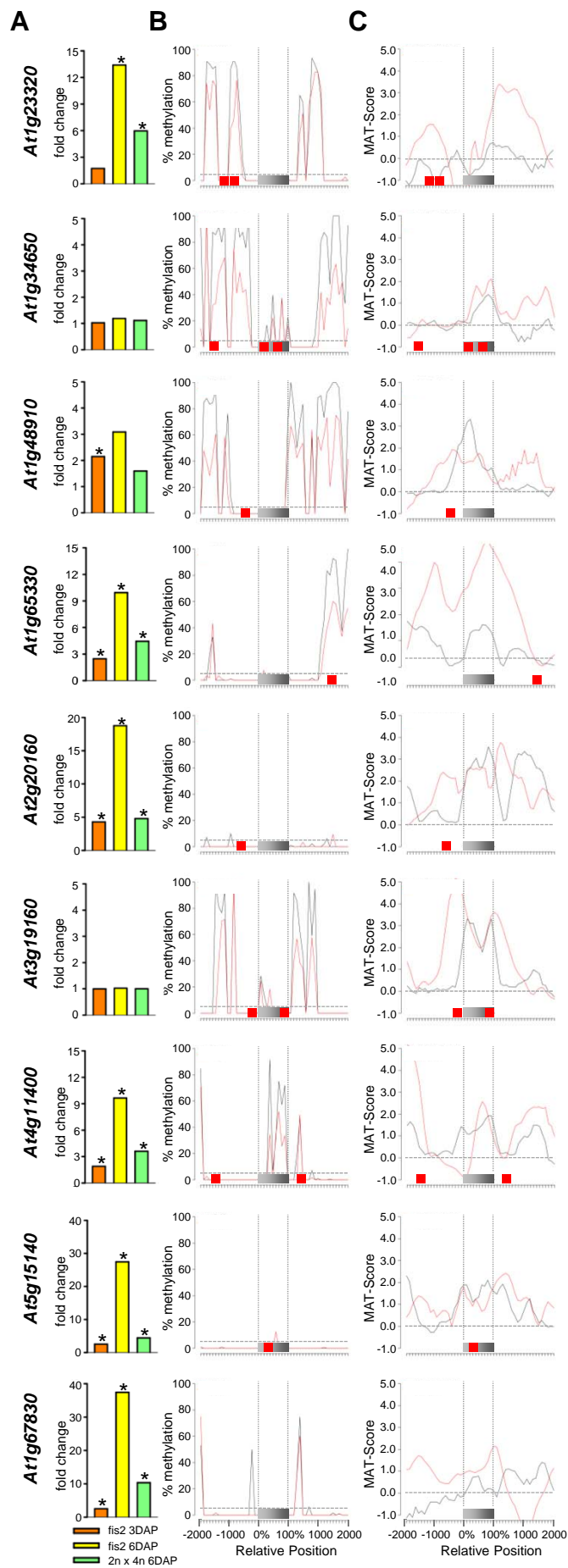

Supplement: Figure S4 — CG DNA Methylation Profiles of PEGs in Vegetative Tissues and Endosperm and Expression Analysis of PEGs in fis2 and 2n×4n Interploidy Crosses. (A) Fold-changes of PEG expression in fis2 mutant seeds at 3 and 6 days after pollination (DAP) and from seeds derived from pollination with tetraploid pollen donors at 6 DAP compared to wild-type seeds at the corresponding time points. Data are based on ATH1 microarray signals after RMA normalization. Significantly deregulated genes are marked by an asterisk. (B) CG DNA methylation profiles of indicated PEGs in vegetative tissues (black line) or endosperm (red line) based on data published by [7], [41]. The gray bar represents the annotated gene body from transcription start (left) to transcription end (right). Red boxes represent transposable elements. Profiles are shown for 5% length intervals along the gene body and for 100 bp sequence intervals for the 2-kb regions upstream and downstream of each gene. The vertical dotted lines mark the gene body. The horizontal dashed line marks the DNA methylation level in vegetative tissues of TAIR8-annotated genes at the transcriptional start site. (C) H3K27me3 profiles of indicated PEGs in vegetative tissues (black line) or endosperm (red line) based on data published by [52], [64]. The gray bar represents the annotated gene body from transcription start (left) to transcription end (right). Red boxes represent transposable elements. Profiles are shown for 5% length intervals along the gene body and for 100 bp sequence intervals for the 2-kb regions upstream and downstream of each gene. The vertical dotted lines mark the gene body. The horizontal dashed line marks the H3K27me3 level of TAIR8-annotated genes at the transcriptional start site. (PDF) [file pgen.1002126.s004.pdf]

**Figure S5**

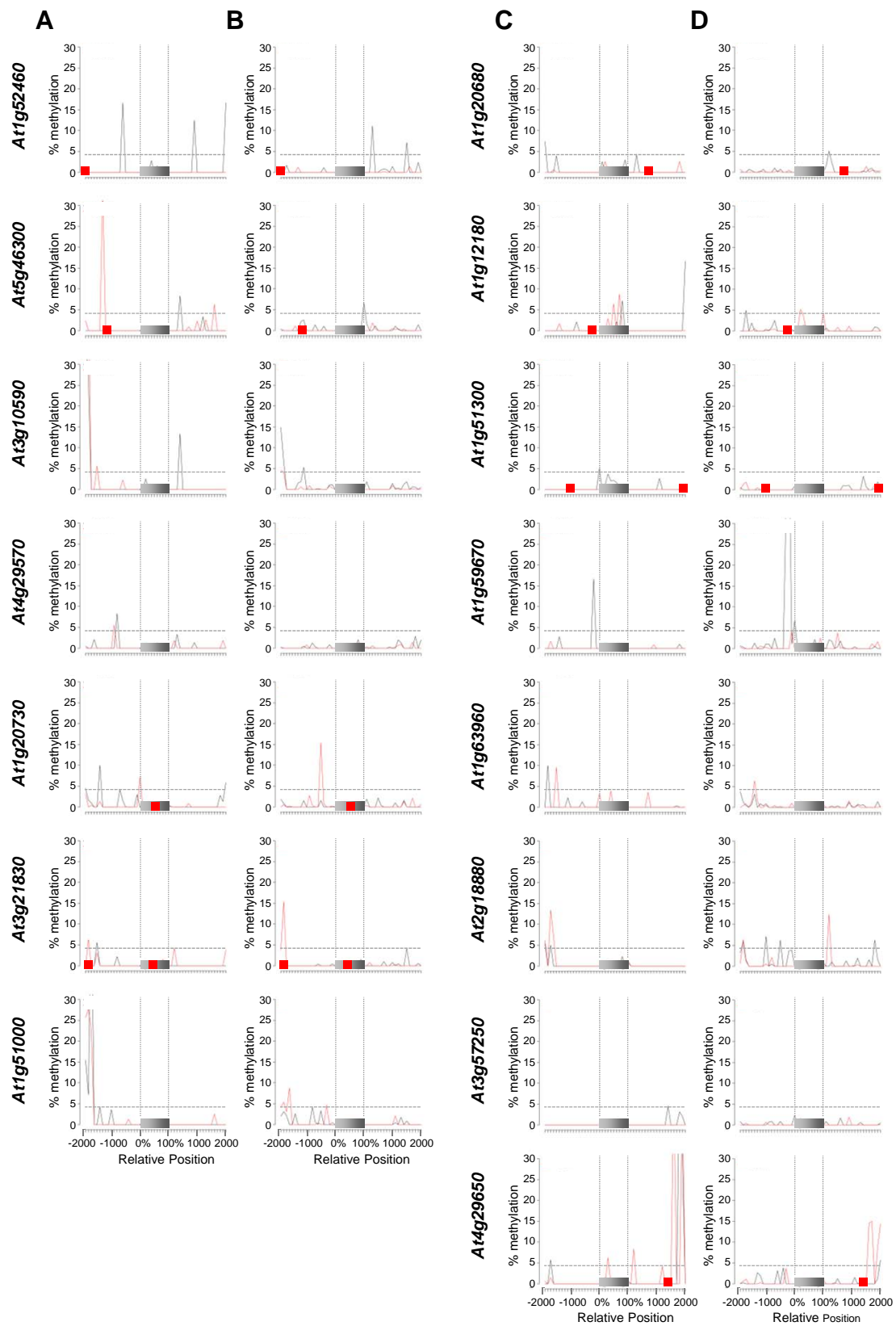

Supplement: Figure S5 — CHG and CHH Methylation Profiles of MEGs. (A, B) CHG (A) and CHH (B) DNA methylation profiles of MEGs shown in Figure 4 in vegetative tissues (black line) or endosperm (red line) based on data published by [7], [41]. (C, D) CHG (C) and CHH (D) DNA methylation profiles of MEGs shown in Figure S3A in vegetative tissues (black line) or endosperm (red line) based on data published by [7], [41]. The gray bar represents the annotated gene body from transcription start (left) to transcription end (right). Red boxes represent transposable elements. Profiles are shown for 5% length intervals along the gene body and for 100 bp sequence intervals for the 2-kb regions upstream and downstream of each gene. The vertical dotted lines mark the gene body. The horizontal dashed line marks the DNA methylation level in vegetative tissues of TAIR8-annotated genes at the transcriptional start site. (PDF) [file pgen.1002126.s005.pdf]

Figure S6

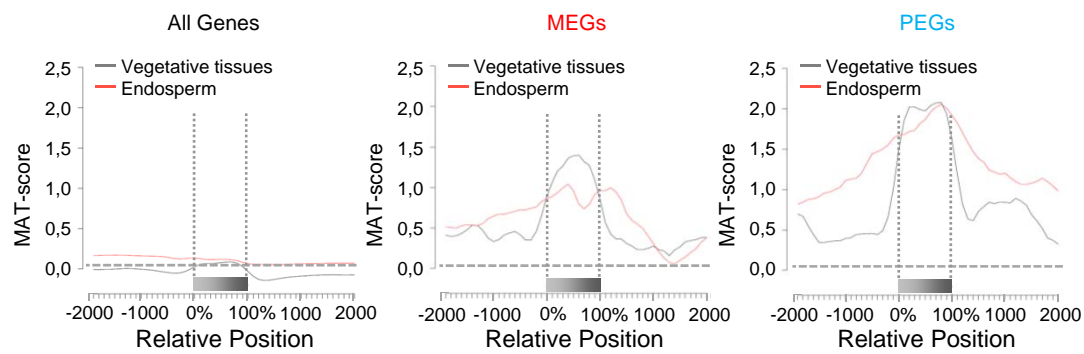

Supplement: Figure S6 — Average H3K27me3 profiles of vegetative tissues and endosperm. Average H3K27me3 profiles of vegetative tissues (black line) or endosperm (red line) of TAIR8-annotated genes (left panels), MEGs (middle panels), and PEGs (right panels). MEGs and PEGs correspond to all genes indicated in Tables S4 and S7, respectively. The gray bar represents the annotated gene body from transcription start (left) to transcription end (right). Profiles are shown for 5% length intervals along the gene body and for 100 bp sequence intervals for the 2-kb regions upstream and downstream of each gene. The vertical dotted lines mark the gene body. The horizontal dashed line marks the H3K27me3 level of TAIR8-annotated genes at the transcriptional start site. (PDF) [file pgen.1002126.s006.pdf]

Figure S7

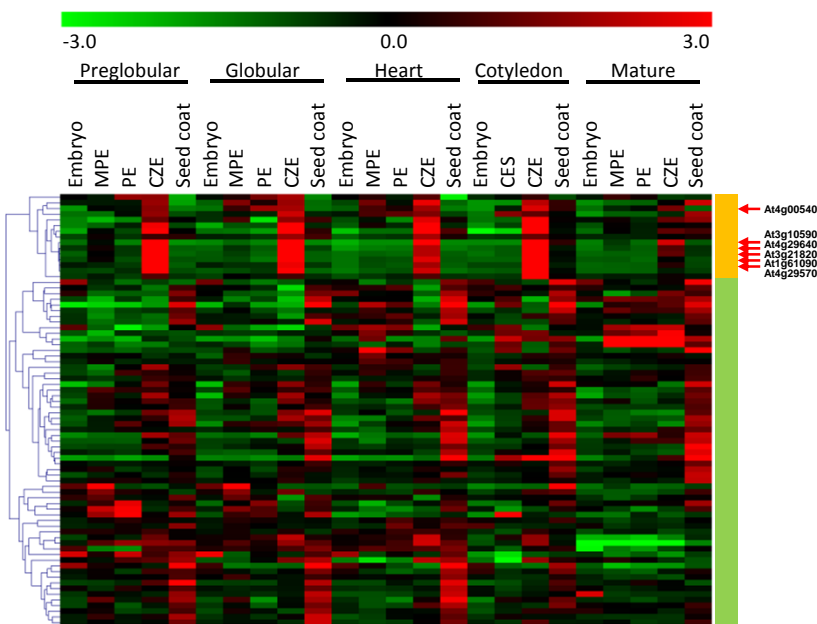

Supplement: Figure S7 — Identification of windows containing significantly enriched numbers of clustered MEGs (A) and PEGs (B). The significance threshold (p = 0.05) is indicated by a red line. (PDF) [file pgen.1002126.s007.pdf]

Figure S9

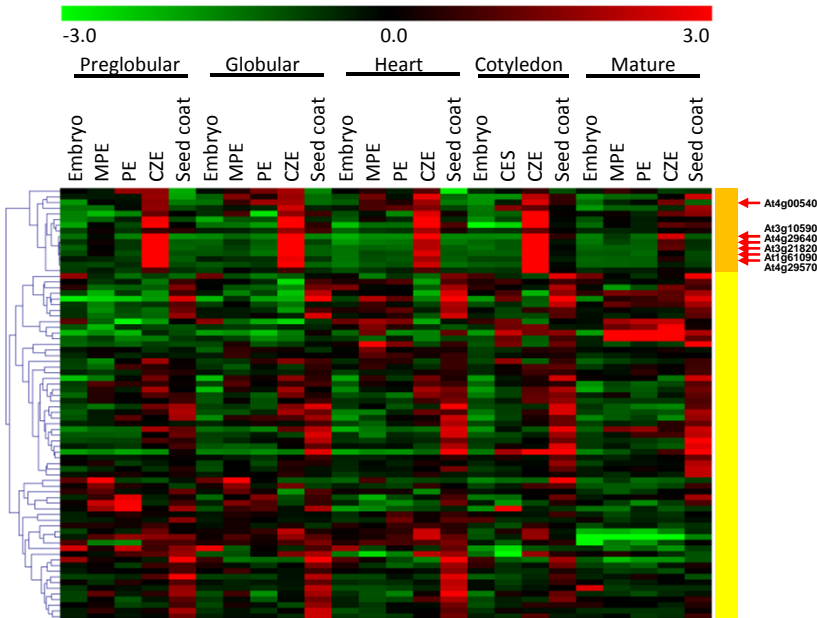

Supplement: Figure S9 — Cluster analysis of MEGs identified by [45] that overlap with unfiltered MEGs identified in this study (Table S15). Cluster analysis of MEGs was based on their expression in embryo, endosperm and seed coat during different stages of seed development. The cluster containing genes with low or without expression in seed coat is marked by a vertical orange bar. The cluster containing genes with high expression in the seed coat is marked by a vertical yellow bar. Genes present in our filtered MEG dataset (Table S7) are indicated. Each row represents a gene, and each column represents a tissue type. Tissue types are: embryos from the preglobular stage to the mature stage, micropylar (MPE), peripheral (PE) and chalazal (CZE) endosperm derived from seeds containing embryos of the preglobular stage to the mature stage, and seed coat derived from seeds containing embryos of the preglobular stage to the mature stage. Red or green indicate tissues in which a particular gene is highly expressed or repressed, respectively. (PDF) [file pgen.1002126.s009.pdf]
