# Supplementary material for: High-Resolution Analysis of Parent-of-Origin Allelic Expression in the Arabidopsis Endosperm
Source: PLoS Genet. 2011 Jun 16;7(6):e1002126. doi: 10.1371/journal.pgen.1002126 (PMC3116908; doi:10.1371/journal.pgen.1002126)
Supplement: Figure S8 — Overlap of MEGs (A) and PEGs (B) identified by [45] and MEGs and PEGs identified in this study. MEGs_LC and PEGs_LC correspond to MEGs and PEGs identified by [45] using Ler/Col accessions; MEGs_BC and PEGs_BC correspond to MEGs and PEGs identified in this study using Bur-0/Col-0 accessions. Unfiltered MEGs_LC and PEGs_LC were identified by [45] using p≤0.001 and p≤0.05, respectively. Unfiltered MEGs_BC and PEGs_BC correspond to data shown in Tables S1 and S2, respectively. Filtered MEGs_BC and PEGs_BC correspond to data shown in Tables S4 and S7, respectively. (PDF) [file pgen.1002126.s008.pdf]

Figure S8

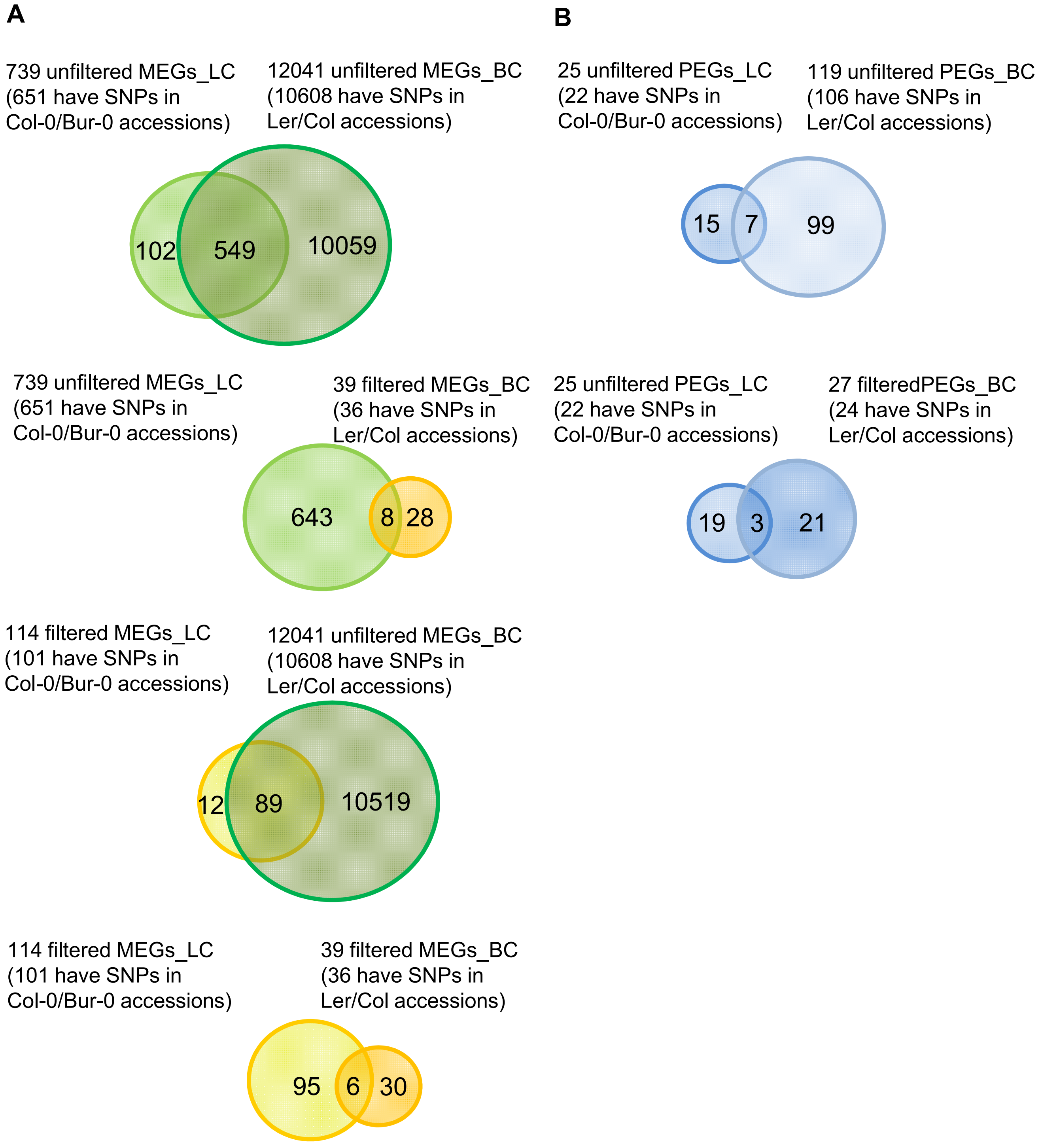
A
B
739 unfiltered MEGs_LC (651 have SNPs in Col-0/Bur-0 accessions)
12041 unfiltered MEGs_BC (10608 have SNPs in Ler/Col accessions)
102
549
10059
25 unfiltered PEGs_LC (22 have SNPs in Col-0/Bur-0 accessions)
119 unfiltered PEGs_BC (106 have SNPs in Ler/Col accessions)
15
7
99
739 unfiltered MEGs_LC (651 have SNPs in Col-0/Bur-0 accessions)
39 filtered MEGs_BC (36 have SNPs in Ler/Col accessions)
643
8
28
25 unfiltered PEGs_LC (22 have SNPs in Col-0/Bur-0 accessions)
27 filteredPEGs_BC (24 have SNPs in Ler/Col accessions)
19
3
21
114 filtered MEGs_LC (101 have SNPs in Col-0/Bur-0 accessions)
12041 unfiltered MEGs_BC (10608 have SNPs in Ler/Col accessions)
12
89
10519
114 filtered MEGs_LC (101 have SNPs in Col-0/Bur-0 accessions)
39 filtered MEGs_BC (36 have SNPs in Ler/Col accessions)
95
6
30
