# Supplementary material for: High-Resolution Analysis of Parent-of-Origin Allelic Expression in the Arabidopsis Endosperm
Source: PLoS Genet. 2011 Jun 16;7(6):e1002126. doi: 10.1371/journal.pgen.1002126 (PMC3116908; doi:10.1371/journal.pgen.1002126)
Supplement: Table S8 — GO analysis of MEGs and PEGs (including accession-dependent MEGs and PEGs). (PDF) [file pgen.1002126.s017.pdf]

Table S8. GO analysis of MEGs and PEGs (including accession-dependent MEGs and PEGs).

MEGs

Biological Process

GO term	p-value	Number	Definition
GO:0009056	0.0012	7	Catabolic process
GO:0006355	0.0022	6	Regulation of transcription, DNA-dependent
GO:0032774	0.0027	6	RNA biosynthetic process

Molecular Functions

GO:0004126	2,87E+07	3	Cytidine deaminase activity
GO:0003700	0.043	6	Transcription factor activity

PEGs

Biological Process

GO:0006996	0.012	3	Nucleus organization
GO:0032502	0.020	5	Developmental process
