# Supplementary material for: High-Resolution Analysis of Parent-of-Origin Allelic Expression in the Arabidopsis Endosperm
Source: PLoS Genet. 2011 Jun 16;7(6):e1002126. doi: 10.1371/journal.pgen.1002126 (PMC3116908; doi:10.1371/journal.pgen.1002126)
Supplement: Table S9 — Median omega, dN and dS values as calculated from pairwise alignments between Arabidopsis thaliana and Arabidopsis lyrata orthologs. Measure of spread represented as the semi interquartile range. (PDF) [file pgen.1002126.s018.pdf]

Table S9. Median omega, *d*N and *d*S values as calculated from pairwise alignments between *Arabidopsis thaliana* and *Arabidopsis lyrata* orthologs. Measure of spread represented as the semi interquartile range.

	**Nr. of genes tested**	***d*N/*d*S**	***d*N**	***d*S**
MEGs	27	0.717 ± 0.354	0.106 ± 0.044	0.152 ± 0.028
PEGs	19	0.481 ± 0.255	0.109 ± 0.047	0.192 ± 0.045
Background genes	19,965	0.181 ± 0.101	0.026 ± 0.015	0.146 ± 0.032
