# Supplementary material for: High-Resolution Analysis of Parent-of-Origin Allelic Expression in the Arabidopsis Endosperm
Source: PLoS Genet. 2011 Jun 16;7(6):e1002126. doi: 10.1371/journal.pgen.1002126 (PMC3116908; doi:10.1371/journal.pgen.1002126)
Supplement: Table S12 — Number of homologous genes to MEGs and PEGs (including accession-dependent MEGs and PEGs) in comparison to the genome-wide frequency of gene homologs. (PDF) [file pgen.1002126.s021.pdf]

Table S12. Number of homologous genes to MEGs and PEGs (including accession-dependent MEGs and PEGs) in comparison to the genome-wide frequency of gene homologs.

	Number of homologs	Number of genes	Percentage	p value
Genome	0	6251	23.17	
	>0	20726	76.83	
MEGs	0	4	10.26	0.023
	>0	35	89.74	
PEGs	0	2	7.41	0.026
	>0	25	92.59	

P values were determined using hypergeometric testing.
