# Supplementary material for: High-Resolution Analysis of Parent-of-Origin Allelic Expression in the Arabidopsis Endosperm
Source: PLoS Genet. 2011 Jun 16;7(6):e1002126. doi: 10.1371/journal.pgen.1002126 (PMC3116908; doi:10.1371/journal.pgen.1002126)
Supplement: Table S20 — Primers and enzymes used for allele-specific expression analysis. (PDF) [file pgen.1002126.s029.pdf]

Table S20. Primers and enzymes used for allele-specific expression analysis.

**Locus**	**Primer (5’-3’)**	**Enzyme**
AT1G51000	ATGGCTAATATTGTTGGAAACACT,<br>CTTCTCCTTCACAGTTTCTTT	Ban I<br>(1 site in Bur-0)
AT3G21830	CTGCTTCTTCCTCAGGTGTT,<br>TGATCTCGAGAAGTGGGACA	Hinf I<br>(1 site in Bur-0)
AGL36	AGTCATTTTCCGACTCTCCTATTCATG,<br>GGTAGTTGATTGGATGGTAGTTGATG	TspR I<br>(1 site in Bur-0)
AGL96	GTGAACTTGCTGATCTCTCCCC,<br>GCTCTCTCCTTTATAGGTCCCCAA	Hph I<br>(1 site in Bur-0)
AGL28	GGTAAAAATGACCAACGAATCAAAC,<br>TGGACTATTGACTGCTTGGTCTCTT	Bsr I<br>(1 site in Bur-0)
AT1G35183	ATGGAAAGTAACAACTTCATTCGTG,<br>TCAGAACCTGTCTCCATATCATCG	Nla III<br>(1 site in Col-0)
AT1G49290	AGGGAGAAACAATTGGCTTTAGA,<br>CTATCACTTCCCCGAATCTCCTA	Fok I<br>(1 site in Col-0)
AT4G31900	GAAAACCAATGTAATCCTTGCG,<br>GAAAATGCATCAGAGCGAAAAG	detection by<br>sequencing
AGL23	TACACAAAGCTTCATGTTCAAATGC,<br>ACCCCTGGTTTGTGGAGATATTA	Apa I<br>(1 site in Bur-0)

AT5G54350	AGCCTCCTGTTGCTCCTCCT,<br>AACCACCTTATCCTCCCGATAG	Tsp45 I<br>(1 site in Col-0)
At3G50720	GTTACCGAACTCGTAAGAGGTGG,<br>CCAATTCGGAGTGGCTCA	Ava I<br>(1 site in Bur-0;<br>2 sites in Col-0)
At1g52460	AATGGATATGTCCATCTCTCGTGCT,<br>CGTTTTAGGCTCTTCGCCTT	detection by<br>sequencing
At3g23060	TGAAGACAATGGATTGGACGAA,<br>TCTTTTCCACCGGTTTCTTACC	detection by<br>sequencing
At5g03020	ATACATAATCGGTGGATTCGTTAGG,<br>GCATGCCTTGGTCGTAAAGTAAT	detection by<br>sequencing
At1g60970	AACAAATGCTAGGACCGAAGTGG,<br>TTAGGGTCAGTGCTATTGATCCC	detection by<br>sequencing
At2g19400	GCCAGTGGTCAATGGAGAACTT,<br>AATTGCAGAGTCACTTCGGGTAG	detection by<br>sequencing
At4g29570	TCTCCGGATCACCTGTCGTAG,<br>ATGTTCCTTTCTGACCGGTTG	detection by<br>sequencing
At5g46300	TTGGAGAAAAAGAGATTGACGATC,<br>GACTCCTTTTCTCCAATGAACACTT	detection by<br>sequencing
At3g10590	CCAACGATGTTAATCAGATGCC,<br>GCCCTCGGTAGACTCCAAAGTT	detection by<br>sequencing
At1g20730	CTCACCAGGCAGATCGCAA,<br>ACCGCAGTGAGAGCAGAGATTA	detection by<br>sequencing

At1g60400	ATCAGAAATTTTCTCACCGGGA,<br>TGAAACTGGTGTCTTCTGCTGAAC	detection by sequencing
At5g50470	GCGACGTTGTCCCAAGAGA,<br>TCCAATTTCCCCAGCTGC	detection by sequencing
At3g62230	TAACGACCGGTTAATGAAGTTGG,<br>GCATGAATCTGATTCCAACAAACTC	detection by sequencing
At3g49770	AGGATTCTCCCTACAACCTTCTACA,<br>GATTATATGCATCATCCTCGTCG	detection by sequencing
At1g66630	ACACTGTTGGAGCTTGATCTTCTG,<br>TGGTTCAGGGCAGTAGCAAAG	detection by sequencing
At1g11810	GCAAAAACCGTAACCACAAAATC,<br>TCATCACCCAAAGACTCAAACG	detection by sequencing
At4g11940	AAAGTTCTTCTTTGCTTGGTCGC,<br>TCATACCATCCAAACCGATTGT	detection by sequencing
